# DIY Virtual Chemical Libraries - Novel Starting Points for Drug Discovery

**DOI:** 10.1021/acsmedchemlett.3c00146

**Published:** 2023-08-30

**Authors:** Gergely Takács, Dávid Havasi, Márk Sándor, Zsolt Dohánics, György T. Balogh, Róbert Kiss

**Affiliations:** † Department of Chemical and Environmental Process Engineering, Faculty of Chemical Technology and Biotechnology, Budapest University of Technology and Economics, Műegyetem rakpart 3, Budapest 1111, Hungary; ‡ Mcule.com Kft, Bartók Béla út 105-113, Budapest 1115, Hungary; § Department of Pharmaceutical Chemistry, Faculty of Pharmaceutical Sciences, Semmelweis University, Hőgyes Endre utca 7-9, Budapest 1092, Hungary

**Keywords:** chemical space, library, drug discovery, novelty

## Abstract

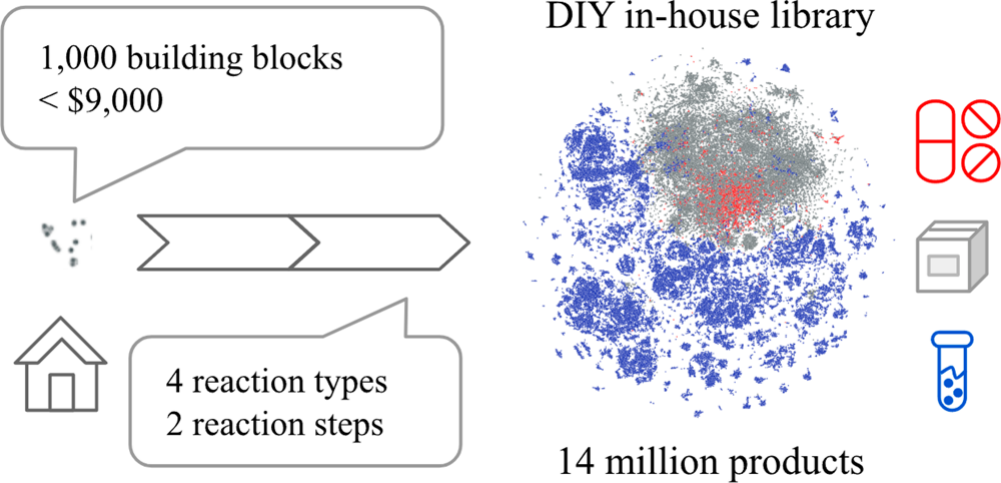

The advancement of *in silico* technologies such as library enumeration and synthetic feasibility prediction has made drug discovery pipelines rely more and more on virtual libraries, which provide a significantly larger pool of compounds than in-stock supplier catalogs. Virtual libraries from external sources, however, may be associated with long delivery time and high cost. In this study, we present a Do-It-Yourself (DIY) combinatorial chemistry library containing over 14 million almost completely novel products built from 1000 low-cost building blocks based on robust reactions frequently applied at medicinal chemistry laboratories. The applicability of the DIY library for various drug discovery approaches is demonstrated by extensive physicochemical property, structural diversity profiling, and the generation of focused libraries. We found that internally built DIY chemical libraries present a viable alternative of external virtual catalogs by providing access to a large number of low-cost and quickly accessible potential chemical starting points for drug discovery.

The application of novel, diverse, and quickly accessible chemical screening libraries in early phase drug discovery is essential for the identification of suitable chemical starting points and for effective hit-to-lead optimization. A large variety of compound libraries exists for drug discovery. Some of the most important aspects that a drug discovery group should consider when selecting chemical libraries are novelty, cost, and acquisition time of the molecules.

Pharmaceutical companies frequently build an in-house inventory of screening compounds. While this approach provides the shortest sample preparation time for physical screening and the highest availability rate (as only a few degraded samples may become unavailable for physical screening), the large upfront investment and ongoing costs of acquiring, maintaining, and managing physical libraries of 100,000 or million members makes this approach less feasible and sustainable for academic research groups and startups. Furthermore, the novelty of the library may be low if the samples are acquired from public supplier catalogs, or it is expensive to maintain if they are sourced from contract research organizations on an exclusive basis.

A different strategy is to acquire compounds from chemical supplier in-stock screening catalogs just before experimental screening. The compounds to be purchased can be selected based on virtual screening results; thereby, only the most promising virtual hits are acquired. Since the number of obtained samples is low, this approach is much more cost-effective. It does not require an upfront investment of large libraries, and the maintenance costs are marginal. Nevertheless, the lack of novelty in suppliers’ public catalogs presents a risk in intellectual property management. The acquisition time of the samples may also highly vary due to the different locations of the supplier warehouses. Furthermore, the availability rate may also diverge by suppliers and catalogs. The management of multiple suppliers can also present a challenge, additional cost, and time; however, these may be overcome by the application of a chemical marketplace providing a one-stop-shop of all supplier catalogs.^1,2^

Alternatively, one may rely on external suppliers’ virtual screening libraries. Some virtual libraries can be highly valuable for theoretical studies, such as GDB-17,^3^ where the molecules are formed by up to 17 non-hydrogen atoms and are connected in accordance with their valence restrictions. There are other—yet smaller—databases that focus on the collection of structures that were physically available once; hence, their synthetic feasibility has been confirmed.^4,5^ The application of such libraries for screening purposes, however, is less practical, as when researchers find an interesting hit, it has to be decomposed with retrosynthetic tools^6^ or custom synthetic paths have to be developed to acquire a physical sample; plus, there is a chance to run into very complex, low-yield synthetic routes. The application of combinatorial chemistry,^7,8^ on the other hand, has the potential to generate enormously large chemical libraries^9,10^ based on predefined synthetic routes and available, in-stock building blocks. Numerous combinatorial approaches exist that combine building blocks with reaction rules to form new molecules (even in multiple, consecutive reaction steps), designed by either academic and commercial groups, that differ primarily in their accuracy of prediction, speed, and validation.^11,12^ Potential hits of such combinatorial libraries can be assigned to a robust, few-step synthetic route, and thus their synthesis can be executed on demand, costs can be precalculated, and the lead time can be kept in an acceptable time frame due to the limited number of applied robust reaction types/steps and to the high availability rate of the reagents. Such virtual libraries typically provide notably higher novelty than in-stock ones simply due to their size (some of the largest of such type libraries can reach the multihundred million or even billion size of unique molecules), exemplified by Mcule ULTIMATE,^11^ WuXi GalaXi,^14^ or Enamine REAL.^15^

Such external supplier virtual catalogs earned significant attention recently as their provided novelty is exceptional, and their delivery success rate and timeline improved significantly compared to earlier released virtual catalogs. Nevertheless their delivery times are still longer, and their pricing is also significantly higher than those of in-stock compounds.

These drawbacks of external virtual libraries might be overcome by building the libraries internally in research groups with synthetic capacity. The design of a relatively large and novel internal virtual chemical library may be built on a small number of low-cost building blocks that can be combined in 1–2 robust reaction steps. The potential advantage of such an approach is that it requires only a small initial investment of low-cost reagents. Due to the low number of necessary chemicals, the associated storage and maintenance costs are marginal compared to those of an internal stock library. The lead time to produce the physical samples of the hits can be kept short if only a few steps of robust reactions are applied. The concept of such internal virtual libraries can be particularly interesting for academic medicinal chemistry research groups with internal synthetic capacity as well as for industrial members who can build up the library either in-house or at an external synthetic partner.

In the present study, we intended to demonstrate the viability of the above concept of building an internal virtual library based on a limited number of low-cost reagents combined by robust reaction rules and the applicability of the library to various drug discovery approaches. The results demonstrate that large, exceptionally novel, and pharmaceutically relevant virtual chemical libraries can be built up from simple reagents. The resulting virtual chemical library might provide an excellent starting point and a useful strategy to those who would like to start a general or a focused drug discovery program (see Figure 1) involving the synthesis and optimization of the most promising compounds. Such do-it-yourself (DIY) in-house virtual screening libraries can be highly valuable tools for medicinal chemistry research groups.

**Figure 1:**
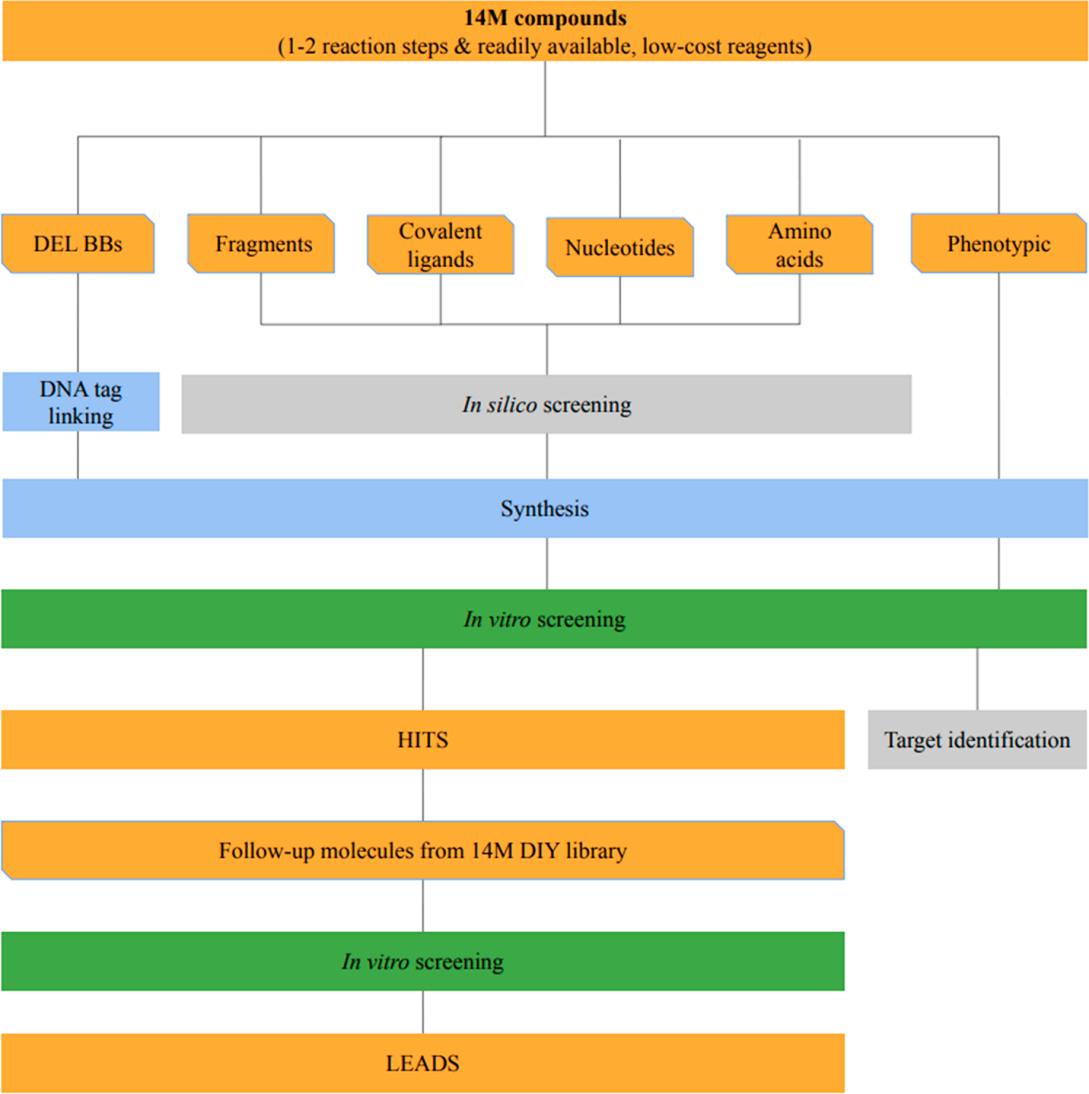
Potential and schematic drug discovery workflows starting from DIY virtual chemical libraries to the selection and synthesis of the hit and lead compounds via various early stage drug discovery methods. Synthesis of compounds (blue), computational chemistry steps (gray), *in vitro* screenings (green), and existing and resulting subsets of the DIY in-house library (orange) are indicated with different colors.

The building blocks involved in this study were collected from more than 100 suppliers included in the Mcule database.^16^ Structural errors can have a large negative impact on the results of *in silico* studies; thus, it can be beneficial to use a curated data set, such as the Mcule database, where registered compounds are already rigorously checked, standardized, and corrected.^17,18^ Commercially available building blocks at a price of less than 10 USD per 1 g were first selected (4448 molecules). To generate the virtual chemical library, we applied two reaction steps using Mcule’s enumeration algorithm ARCHIE^11^ with standard settings. The potential chemical reactions were handed over to the enumerator algorithm as reaction SMARTS (SMIRKS)^11,19^ patterns. The algorithm then checked whether any of the favored *main reaction* SMIRKS patterns and none of the unfavored *side reaction* patterns match (i.e., whether the reaction is possible). Finally, the SMILES^20^ representation of the product was generated. In the first reaction step, both reagents were selected from the original reagents set. In the subsequent second reaction step, all the intermediates generated by the first step were allowed to react with any of the original reagents. This process yielded products formed by three reagents in two consecutive reaction steps. Initial evaluation of the DIY building block set revealed that some of the most common reactive functions of the building blocks include amino-, hydroxy-, aryl halide, and carboxylic acid groups. Consequently, four main reaction categories were selected for the library enumeration that are frequently applied by medicinal and synthetic chemistry laboratories^21^ and utilize the above functionalities.

- amide bond formation,^22,28^ the most common reaction type for which several mild reaction conditions are available.
- ester formation between acids and alcohols,^23,24^ which utilizes coupling agents and conditions similar to amide bond formation.
- reactions of heteroaromatic halides and nucleophiles, such as amines, alcohols, or thiols (S_N_Ar—requiring an aromatic ring substituted by a halogen at an electron-poor location,^25^ with a simple base for the reaction; or Buchwald-Hartwig-type reaction that require catalytic systems^26−28^).
- catalytic carbon–carbon couplings including *Suzuki-Miyaura reaction* that is performed between aryl halides or pseudohalides and organoboranes forming a single C–C bond,^29^*Sonogashira coupling* between sp^2^ halides or pseudohalides and a terminal alkyne to form a triple C–C bond,^30^*Heck reaction* between sp^2^ halides or pseudohalides and olefins that form a double C–C bond^31^ between the reactants.

While there is a large range of widely applied reaction types that could be used for building a DIY in-house virtual library (e.g., a wider range of Buchwald-Hartwig couplings, reductive aminations, Wittig condensation, or the formation of various heterocycles),^32^ in the present study our aim was to demonstrate the applicability of the concept on a selected set of reactions and building blocks. There is great potential to increase the size of the generated libraries by extending the set with novel building blocks and reaction types.

The required characteristics of the reagents for the above reactions were defined as SMARTS^19^ patterns which were being matched pairwise. If a match was found, it was further checked against more than 100 other so-called “side reaction” patterns to avoid competitive reactions and byproducts. If only one match was found and that was among our main reactions, the two molecules were allowed to form the product by the algorithm. Cases were allowed where the different conditions (temperature range, reaction time) and additives (acids, bases, etc.) required for the reaction types excluded the possibility for the side reaction to occur, as a built-in functionality of the ARCHIE algorithm. For example, in the case of amidation as a main reaction, Suzuki coupling is not considered as a side reaction (due to the lack of necessary catalyst), while aliphatic halides are capable of forming byproducts. Similarly, some labile substructures are defined to avoid, for example, the decomposition of some esters.

2061 reagents out of the 4448 have turned out to be reactive in at least one of the selected reaction types. To identify the 1000 most efficient reagents yielding the highest number of products at the lowest total price, we used an iterative method. At each iteration, the “Reaction score” of each building block was calculated as the number of potential end-products derived from the building block divided by its price. The reagent with the lowest score was excluded after each iteration. The iterative process was carried out by a python script starting with the above selected 2061 building blocks yielding over 21 million synthesizable products. After the last iteration, the best scoring 1000 building blocks were kept associated with 14,020,690 synthetically feasible products. The cheapest building block had the price of 4.5 USD while the average and median prices were 8.5 and 9.0 USD, respectively. Depending on the price of the building blocks and the number of applicable synthesis steps, the total building block costs range from 9 to 30 USD.

For the 14 million synthetically accessible products generated above, we calculated the following physicochemical properties^33−36^ relevant to classification of drug discovery application using Openbabel^37^ on a 24/48 core machine with AMD EPYC 7401P CPU for 358 min: molar mass, log *P*, polar surface area (PSA), number of H-bond acceptors and donors (*N*_HBA_, *N*_HBD_), number of rotatable bonds (*N*_rotb_), heavy atom count (HAC), number of rings, fraction of sp^3^ carbons, refractivity, number of aromatic rings, number of aliphatic rings, number of chiral centers, acidic group count, basic group count, acidic plus basic group count, noncyclic amide count, O and N atom count, heteroatom ratio.

During the generation of custom libraries the reactive sites and substructures were identified using custom written SMARTS patterns of the respective formulas and matched using Openbabel and Indigo^37,38^ SMARTS matching tools.

Exact matching of DIY in-house products against ChEMBL^17^ molecules was carried out based on the InChI^39^ representations of the products.

DIY in-house products were classified into appropriate libraries based on their property profiles, reactive functionalities, and special substructures. The assigned focused libraries included molecules for DNA encoded library (DEL) design,^40^ covalent warheads,^41,42^ fragment-based drug discovery^35,43^ (FBDD), bioactive compounds suitable for phenotypic screening,^44^ or various derivatives of nucleotides^45,46^ or amino acids.^47,48^ In the case of the amino acid derivative subset, glycine derivatives were excluded since they would have outnumbered every other derivative due to their simple substructure. The detailed criteria on physicochemical properties, desired functional groups, and substructures are collected in Supporting Information Tables S1 and S2, respectively.

The t-distributed stochastic neighbor embedding (t-SNE) algorithm was used for clustering and visualization.^49^ The molecules were described with their extended-connectivity fingerprints (ECFP4)^50^ using the Chemplot python library.^51^ The t-SNE algorithm defines a probabilistic distribution of the molecules in the high-dimensional fingerprint space and then defines a similar probability distribution in the low-dimensional space. In the last step, it minimizes the Kullback–Leibler divergence between the two distributions and results in a two-dimensional (2D) projection of the high-dimensional space. In that projection, we can state that structurally similar objects of the high-dimensional space will appear close to each other in the low-dimensional space. The algorithm was initialized with random parameters, and perplexity was set to 70.

In the present study, only a small portion of the most common and robust reaction types was used which do not require extreme reaction conditions, extensive optimization, or rare and expensive additives. These reactions included amide and ester bond formation protocols,^22,23,23,24^ catalytic C–C couplings,^29−31^ and S_N_Ar^25^ or Buchwald-Hartwig-type reactions^26−28^ that are easily performed in any organic chemistry laboratories; thus, hits derived from the library can be obtained in a “Do-It-Yourself” (DIY) fashion. The ARCHIE^11^ enumerator algorithm applied in this study already implements the synthetic rules of the above robust chemical reactions as well as various side-reaction rules.

The building block set of the DIY in-house library, derived from the Mcule database, contains 4448 potential reagents priced below 10 USD/1 g. Only 2061 out of the 4448 building blocks have shown reactivity in at least one of the reactions. 349,948 synthetically accessible products were generated in the first reaction step using the aforementioned reaction types, while the second reaction step resulted in 21,823,447 additional products.

To further optimize the cost of the building blocks, the top 1000 reagents with the highest number of end-products per USD were iteratively selected. As the building blocks with the lowest reactivity scores were excluded, the number of products and the overall budget was reduced.

This selection method was effective for eliminating the most expensive building blocks with low combinatorial chemistry potential, as shown in Figure 2. The inflection point of the curve was found to be close to 1000 building blocks (above this point, the number of new products gained per invested USD started to decline significantly) (Figure 2).

**Figure 2:**
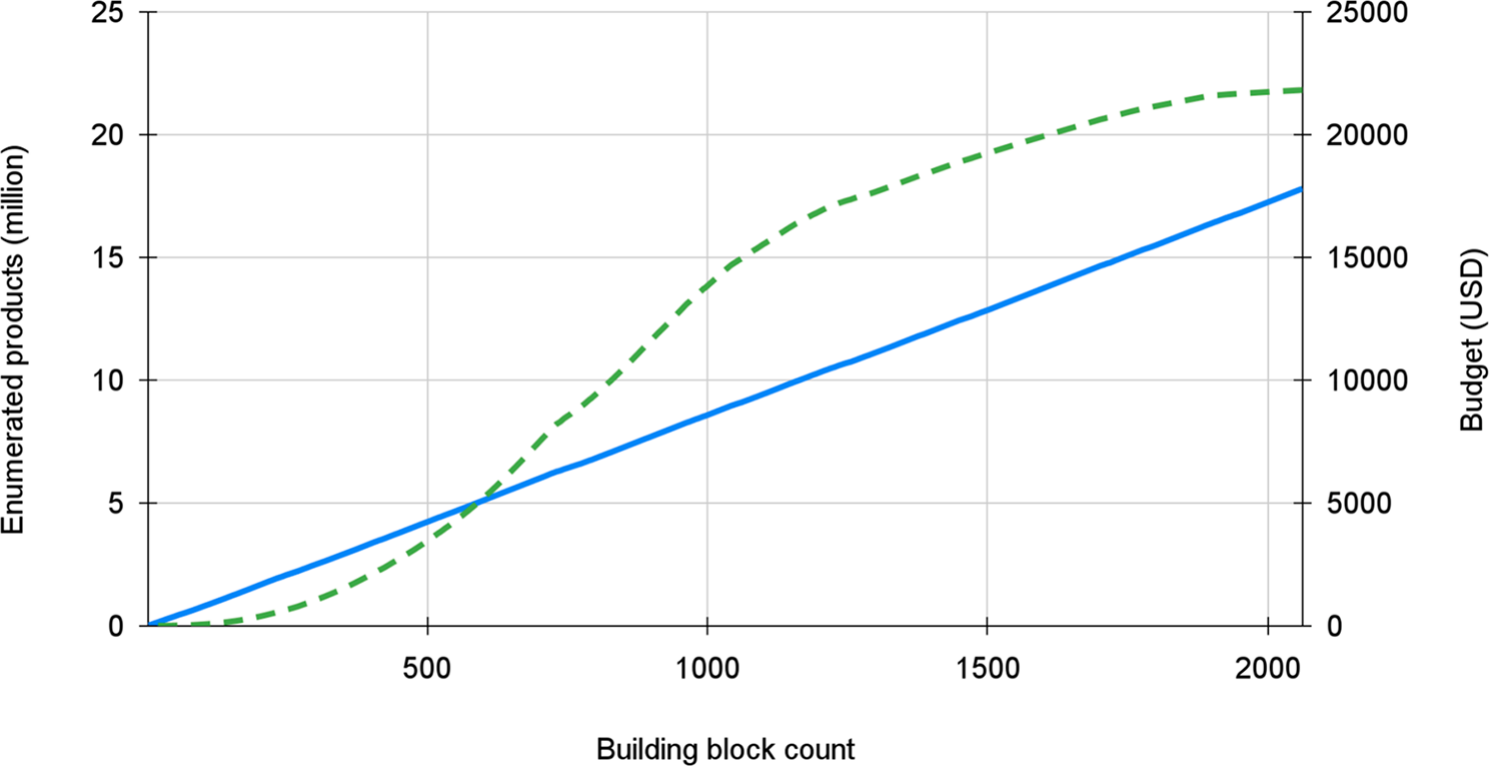
Effect of the building block count on the number of enumerated products (green dashed line in million products on the left *y*-axis) and on the total budget (blue line in USD on the right *y*-axis). During the selection (building block elimination) process we continuously moved from the right to the left along the *x*-axis. The final selection of 1000 building blocks is close to the inflection point of the green (enumerated product) curve.

The best-scoring 1000 building blocks yielded 14,020,690 products, comparable in size to the currently available full in-stock chemical space, for an estimated building block price of 8576 USD. The estimated cost of a 2-step synthesis product includes the price of the building blocks (3 times 8.6 USD), coupling agents or catalysts (estimated as 0.1 to 2.5 USD per reaction, e.g., 10 mg XPhos PD G3), and solvents (∼1 USD). Altogether, the overall direct costs fall well short of the price of the virtual compounds.

The contribution of the applied reaction types to the first and second reaction steps are summarized in Table 1. The first step is dominated by amide formation, while the second step mainly consists of C–C couplings.

**Table 1:** Number of Products Formed by Each of the Four Distinct Reaction Types Per Each Reaction Step

Reaction type	1st step	2nd step
Amide formation	5,032,518	4,505,054
Ester formation	3,137,944	502,208
C–C coupling	1,385,644	6,455,318
S_N_Ar	4,464,584	2,379,971

Analyzing the novelty of the database we found that 13,774,903 products (98.2%) have not been published in patents or research articles yet (no matching item found in PubChem,^4,31^ Mcule, Enamine, ZINC,^5^ ChEMBL,^17^ and SureChEMBL^17,52^ databases), nor are they currently available from commercial sources like Mcule, Enamine, and ZINC.^5^ The unexpectedly small overlap between the DIY in-house library and the currently available public databases suggests a large gap between the available and already exploited parts of the synthetically feasible chemical spaces. Thus, there is great potential to exploit such portions of the chemical space for drug discovery.

To validate our forward-predicting synthetic rules and the methodology used for the generation of the DIY in-house library, example products were synthesized for each of our four main reaction types (a total of 10 different compounds were synthesized, from which 4 were intermediates for a second reaction step). From the various reaction conditions available in the literature, we selected those that are robust and commonly applied and that do not require expensive or custom-made additives. The detailed protocols and results are reported in the Supporting Information (Figures S1–S29). A short overview of the validated compounds is shown in Figure 3. Out of the four synthesized compounds, two were found to be completely novel and have not been published or made available in public catalogs, according to PubChem, Mcule, Enamine, ZINC, ChEMBL, and SureChEMBL databases.

**Figure 3:**
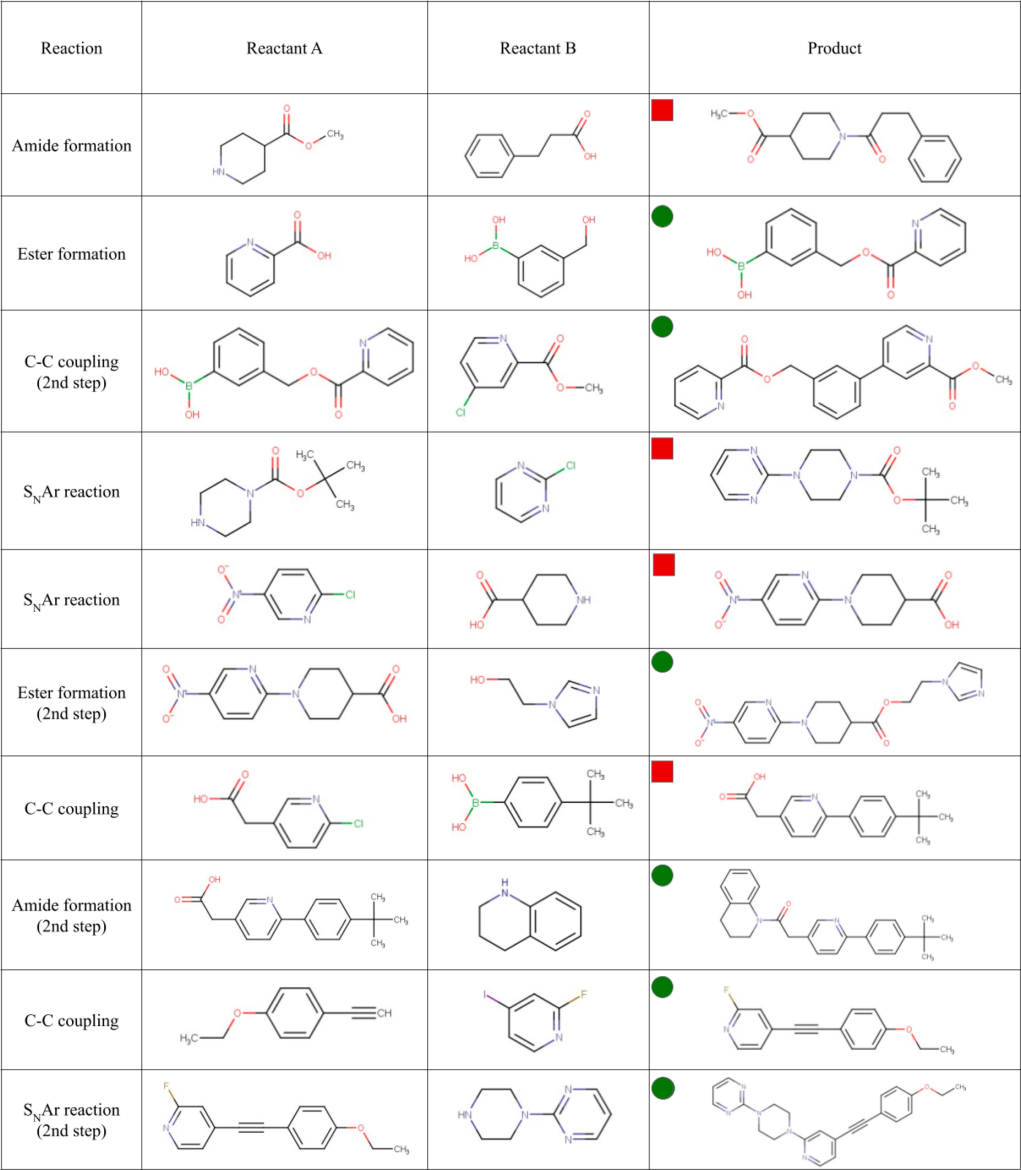
Reaction validations are for the four distinct reaction types. Obtained novel molecules and already published/commercially available ones are labeled by *green dots* and *red squares*, respectively. For detailed synthetic conditions and results, see the Supporting Information.

Additionally, to investigate whether the identified synthetically feasible products are suitable for various drug discovery applications, we characterized them by their most common physicochemical properties.^33,34^

At first glance we set up three subcategories: *Rule of three* compliant^53^ molecules, lead-like molecules according to Oprea et al.,^53,54^ and drug-like molecules based on Lipinski’s *Rule of five*.^33^ We found that 86.4% of the DIY in-house library meets drug-like property limits and 31.6% fulfills leadlike criteria. A rather small portion (0.1%) of the DIY in-house library is suitable for fragment-based screening, which might be reasoned by the fact that fragment chemical space is much smaller than lead-like and drug-like chemical spaces but provides a better sampling. Additionally, the combinatorial design may result in fewer fragment-sized products. The physicochemical characteristics of the DIY in-house virtual chemical library are shown in Figure 4. Subsequently, we classified the molecules of the DIY in-house library into six, more detailed subsets to demonstrate its applicability in various drug discovery strategies beyond classical *in vitro* high-throughput screening: (i) DNA-encoded library synthesis-compatible building blocks, (ii) covalent binders; (iii) nucleotide derivatives, (iv) amino acid derivatives, and (v) bioactive molecules for phenotypic screening. Surprisingly, we found that a significantly larger portion of the DIY in-house library (11.9%) could be applied for the analyzed drug discovery approaches (apart from general purpose virtual screening) than that of the commercially available compounds of over 100 suppliers’ in-stock catalogs (5.6%) (Table 2).

**Figure 4:**
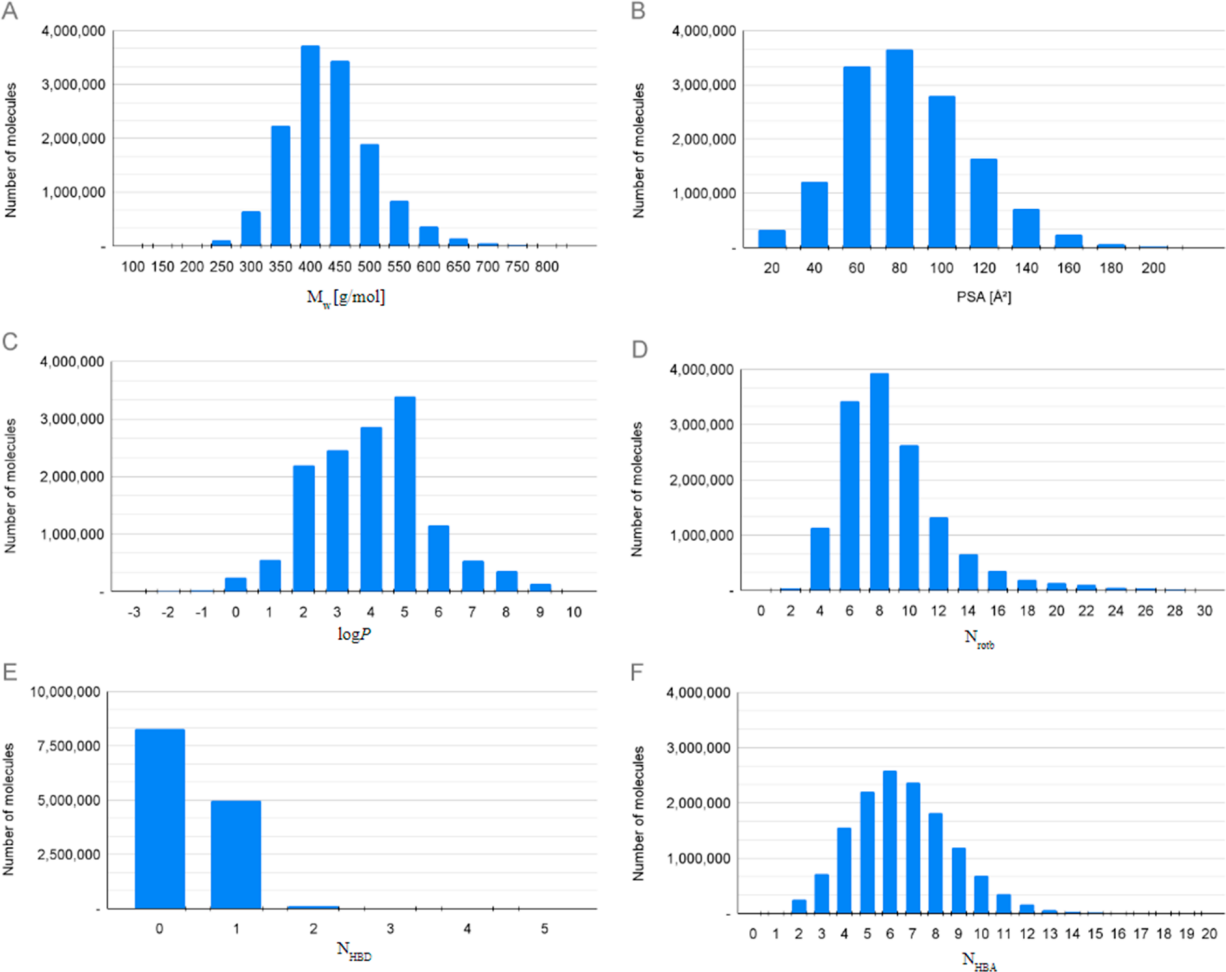
Distribution of molar mass (A), polar surface area (B), log *P* (C), number of rotatable bonds (D), H-bond donors (E), and H-bond acceptors (F) of the 14 M DIY in-house virtual chemical library.

**Table 2:** Number and Ratio of Products in Each Focused Library Subset Selected from the 14M DIY In-House Library of Synthetically Accessible Products and the 8.7M Mcule In Stock Database

**Library**	**Number of DIY library products**	**Number of Mcule In Stock products**
Nucleotide derivatives	17,217 (0.12%)	74,327 (0.86%)
Amino acid derivatives (GLY excluded)	439,959 (3.14%)	30,245 (0.35%)
Covalent warheads (fragment sized)	1148 (0.01%)	41,402 (0.48%)
Covalent warheads (druglike)	167,255 (1.19%)	116,672 (1.34%)
DEL BBs with 2 reaction sites	980,901 (7.00%)	197,242 (2.27%)
DEL BBs with 3 reaction sites	67,699 (0.48%)	7138 (0.08%)
Bioactive molecules	115 (0.00%)	17,146 (0.20%)
General purpose screening library	7,440,227 (53.07%)	3,958,921 (45.60%)

These results are mainly attributed to the high number of amino acid derivatives and DEL applicable building blocks found in the DIY in-house library. The smaller-sized phenotypic subset can be explained by the exceptionally high novelty rate of the DIY library; thus, there is only limited or no information available on these compounds yet. Nevertheless, 115 products of the DIY in-house library already have ChEMBL annotations with sub-micromolar activity. Targets of these bioactivities span kinases, receptors, oxidoreductases, dehydrogenases, and several other substrates.

The subset of products with various covalent warheads was further split into drug-like and fragment-sized categories. The most common functions in these subsets were cyanide, carbamate, and boronic acid functions. Products of the DIY in-house library that can be applied in DNA encoded library (DEL) synthesis can be divided into three subcategories based on the number of reactive functionalities therein (1, 2, or 3). Products with only one reactive functionality (capping building blocks) are omitted in the current classification due to their high prevalence. In addition to the above focused libraries, a general-purpose screening library was prepared—filtered against unwanted reactive sites and PAINS motifs.^55−58^

An overview and visualization of 1000 random molecules from each of the various subsets was generated using t-SNE algorithm^49,50^ with ECFP4^51^ fingerprint descriptors depicted on Figure 5. Interestingly, most subsets contained structurally diverse compounds, while, e.g., the nucleotide library was represented by an isolated island.

**Figure 5:**
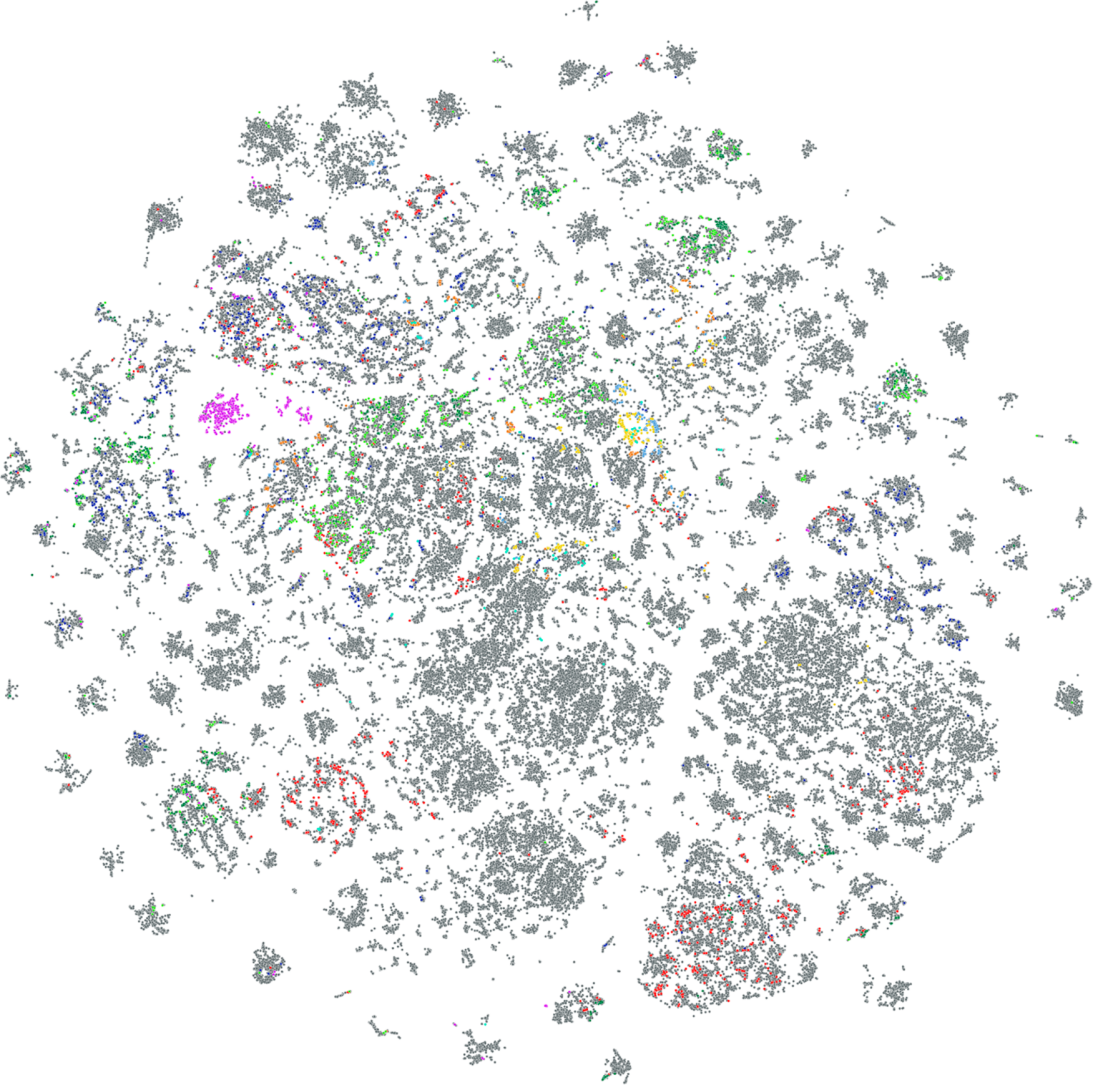
Visualization of drug discovery approach for specific subsets of the DIY in-house library using the t-SNE algorithm. The proximity of points correlates with their structural (ECFP4 fingerprint-based) similarity: full DIY library (gray), amino acid derivatives (red), drug-like covalent warheads (dark blue), fragment-sized covalent warheads (light blue), DEL building blocks with two (dark green) and three (light green) reactive sites, high fps3 (>0.4) Ro3 fragments (brown), low fsp3 (<0.4) Ro3 fragments (yellow), nucleotide derivatives (purple), and known bioactives (turquoise) are shown.

To further analyze the structural characteristics of the DIY in-house library we extended this approach on the Mcule In Stock database as well as on the collection of approved drugs from Drugbank^59^ to see how these reference databases compare to the DIY library. The analysis revealed that the DIY library covers a larger portion of the chemical space, and only smaller parts overlap with the Mcule In Stock database (Figure 6). On the other hand, approved drugs showed more structural similarity to the Mcule In Stock database and vice versa. Nevertheless, the close proximity of the known drugs was densely surrounded by DIY products suggesting yet unexplored and unexploited parts of the known drug-like chemical space.

**Figure 6:**
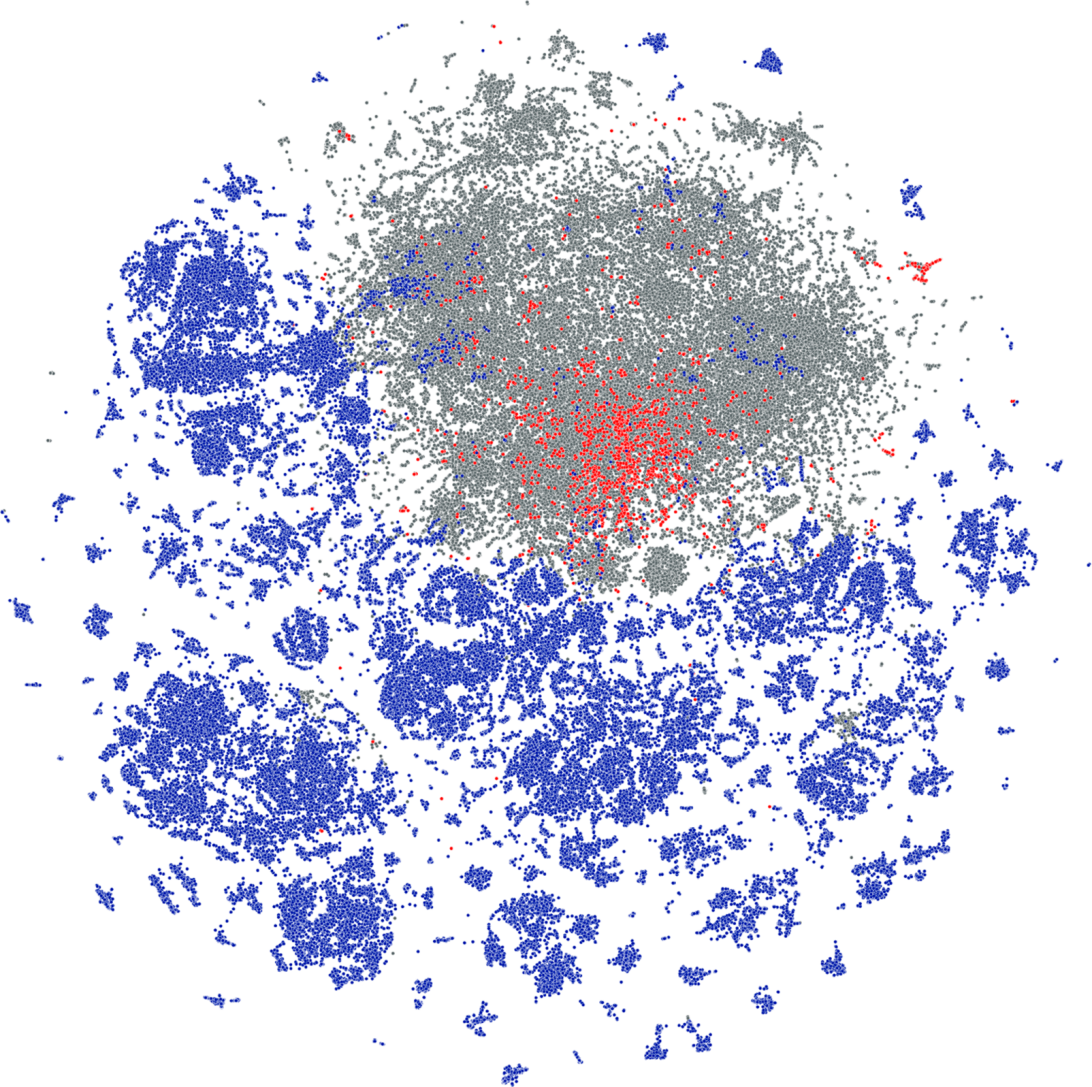
Representation of structural chemical space based on ECFP4 chemical fingerprints of the in-house virtual DIY in-house library (blue), commercial in-stock molecules (Mcule In Stock) (gray), and approved drugs (red).

In this study we showed that a virtual chemical library consisting of 14 million, almost completely novel, synthetically accessible end-products can be built up from only 1000 low-cost building blocks that were selected by an iterative process of maximizing end-product number per cost. Each of these building blocks is available for less than 10 USD in 1 g amount. The application of maximum 2-step, robust reactions ensures quick synthesis and high availability rate. The synthetic feasibility of the DIY in-house library has been demonstrated by synthesizing examples of each applied reaction type. The library has been characterized by the most commonly used physicochemical descriptors with high medicinal chemistry relevance. The property distributions suggested that the majority of the DIY in-house library falls within the drug-like chemical space. Furthermore, based on these descriptors and additional filtering criteria, special focused subsets of the DIY in-house library have been created that are applicable to various drug discovery approaches including DEL, covalent ligand design, and phenotypic screening using bioactive compounds. The DIY in-house library approach offers a comprehensive and easily available cost-effective alternative to medicinal chemistry research groups entering the field of drug discovery or broadening their toolkits to construct their desired focused set of different drug discovery strategies. The DIY in-house virtual chemical library is a low-cost, quickly accessible alternative of in-house and external stock chemical libraries to identify novel, intellectual property free chemical starting points for drug discovery.

## Supporting Information Available

The referenced data for the subset generation and details of the synthesis, used in the current study. Contains the links to the building blocks, the products and the subsets in SMILES format (PDF)

## Abbreviations

DIY: Do-It-Yourself
DEL: DNA encoded library
S_N_Ar: Nucleophilic aromatic substitution
PSA: Polar surface area
HBA: H-bond acceptors
HBD: H-bond donors
rotb: Rotatable bonds
HAC: Heavy-atom count
t-SNE: t-distributed stochastic neighbor embedding
ECFP4: Extended connectivity fingerprint 4
FBDD: Fragment-based drug discovery
