# Supplementary material for: DIY Virtual Chemical Libraries - Novel Starting Points for Drug Discovery
Source: ACS Med Chem Lett. 2023 Aug 30;14(9):1188–97. doi: 10.1021/acsmedchemlett.3c00146 (PMC10510501; doi:10.1021/acsmedchemlett.3c00146)
Supplement: Supplementary file 1 — ml3c00146_si_001.pdf [file ml3c00146_si_001.pdf]

Supplementary Information

DIY virtual chemical libraries - novel starting points for drug discovery

Gergely Takács[1,2], Dávid Havasi[1,2], Márk Sándor[2], Zsolt Dohánics[2], György T. Balogh[3,1,*], Róbert Kiss[2,*]

[1]Department of Chemical and Environmental Process Engineering, Faculty of Chemical Technology and Biotechnology, Budapest University of Technology and Economics, Műegyetem rakpart 3, Budapest, 1111, Hungary
[2]Mcule.com Kft, Bartók Béla út 105-113, Budapest, 1115, Hungary
[3]Department of Pharmaceutical Chemistry, Faculty of Pharmaceutical Sciences, Semmelweis University, Hőgyes Endre utca 7-9, Budapest, 1092, Hungary.

Table S1. Physicochemical property restrictions applied on the covalent subsets. 3
Table S2. Functional groups or substructures required for the corresponding DIY focused subsets. 5
Figure S1. Reaction scheme of amide formation 7
Figure S2. Spectrum of purified amide formation product, retention time of target compound: 3.7 min 7
Figure S3. Mass spectrum of target compound, product ion (M+H+) at 276 m/z 8
Aromatic nucleophilic substitution reaction 8
Figure S4. Reaction scheme of SNAr reaction 8
Figure S5. McLafferty-rearrangement occurring during ionization - target product mass 264 g/mol, rearrangement product mass 208 g/mol 9
Figure S6. Spectrum of purified SNAr reaction product, retention time of target compound: 3.8 min 9
Figure S7. Mass spectrum of the purified product - product ion at 265 m/z (M+H+), McLafferty-rearrangement product at 209 m/z ([M-56]+H+) 9
2-step synthesis with ester formation and Suzuki cross coupling 10
Figure S8. Ester formation reaction scheme 10
Figure S9. Reaction scheme of Suzuki coupling 11
Figure S10. Spectrum of purified 2-step reaction product, retention time of target compound: 1.2 min 12
Figure S11. Mass spectrum of target compound, product ion at 349 m/z (M+H+), dimer ion of product at 697 (M+M+H+) 12
2-step synthesis with SNAr and ester formation 12
Figure S12. Reaction scheme of SNAr reaction 12
Figure S13: Spectrum of purified intermediate, retention time of target at 3.2 min 13
Figure S14: Mass spectrum of target compound, product ion at 252 m/z (M+H+) in positive mode, 250 m/z (M-) in negative mode 13
Figure S15. Reaction scheme of ester formation 14
Figure S16: Spectrum of purified product, retention time of target at 1.2 min 14
Figure S17: Mass spectrum of target compound, product ion at 346 m/z (M+H+) in positive mode 14

2-step synthesis with Suzuki coupling and amide formation 14
Figure S18. Reaction scheme of Suzuki coupling 15
Figure S19: Spectrum of purified intermediate, retention time of target at 1.6 min 15
Figure S20: Mass spectrum of purified intermediate, product ion at 270 m/z (M+H+) in positive mode 15
Figure S21. Reaction scheme of amide formation 16
Figure S22: Spectrum of purified product, retention time of target at 2.2 min 16
Figure S23: Mass spectrum of target compound, product ion at 386 in positive mode 17
2-step synthesis with Sonogashira coupling and SNAr 17
Figure S24. Reaction scheme of Sonogashira coupling 17
Figure S25: Spectrum of purified intermediate, retention time of target at 2.2 min 17
Figure S26: Mass spectrum of intermediate, product ion at 242 m/z (M+H+) in positive mode 18
Figure S27. Reaction scheme of SNAr reaction 18
Figure S28: Spectrum of purified product, retention time of target at 1.9 min 18
Figure S29: Mass spectrum of purified product, product ion at 386 m/z (M+H+) in positive mode 19
Figure S30. The ratio of reported activities of the 88 reported bioactive compounds of the library. **19**

Files

Building block SMILES file - https://doi.org/10.5281/zenodo.7599786
Products SMILES/SDF file - https://doi.org/10.5281/zenodo.7599786
ZIP file containing all the filtered libraries - https://doi.org/10.5281/zenodo.7599786

Tables

	**Covalent warheads (fragment)**	**Covalent warheads (druglike)**
Molar mass	X<300	160<X<450
logP	X<3	-0.4<X<3.5
PSA	-	20<X<120
H-bond acceptors	X<3	2<X<9
H-bond donors	X<3	0<X<2
Rotatable bonds	X<3	2<X<8
Heavy atom count	-	8<X
Rings	-	-
Fsp3	-	0.2<X
Refractivity	-	40<X<130
Aromatic rings	-	X<4
Aliphatic rings	-	1<X
Chiral centers	-	X<2
Acidic group count	-	X<2
Basic group count	-	X<2
Acidic and basic group count	-	X<3
Non cyclic amide count	-	X<1
O and N atom count	-	1<X
Heteroatom ratio	-	0.1<X<1.5

Table S1. Physicochemical property restrictions applied on the covalent subsets.

**Nucleotide**	**Amino acid**	**Covalent warheads**	**DEL building blocks with 2 reaction sites**	**DEL building blocks with 3 reaction sites**
Adenine	Glycine	2-chlor heterocycles	Aldehyde<br>Nitro	Amine<br>Aryl-halide<br>N-Boc-Amino

Cytosine	Alanine	2-cyano heterocycles	Amine<br>Aryl-halide	Amine<br>Aryl-halide<br>Nitro
Guanine	Valine	2-ethynyl heterocycles	Amine<br>Ester	Aryl-halide<br>N-Boc-Amino<br>Carboxylic-acid
Thymine	Leucine	2-vinyl heterocycles	Amine<br>N-benzyl amine	Aryl-halide<br>N-Fmoc-Amino<br>Carboxylic-acid
Uracil	Isoleucine	Acrylamide	Amine<br>N-Boc Amino	Carboxylic-acid<br>Aldehyde<br>Nitro
	Proline	Acrylonitrile	Amine<br>N-Cbz Amino	Carboxylic-acid<br>Aryl-halide<br>Ester
	Serine	Alkylhalide	Amine<br>N-Fmoc Amino	Carboxylic-acid<br>Aryl-halide<br>Nitro
	Threonine	Alkylthiol	Azide<br>Aldehyde	Carboxylic-acid<br>Ester<br>Nitro
	Asparagine	Allenamide	Azide<br>Aryl-halide	Ester<br>N-Fmoc-Amino<br>Carboxylic-acid
	Glutamine	Aziridine	Azide<br>Sulfonyl-halide	N-Boc-Amino<br>N-Cbz-Amino<br>Carboxylic-acid
	Cysteine	Beta lactame	Boronate<br>Aldehyde	Nitro<br>N-Boc-Amino<br>Carboxylic-acid
	Methionine	Beta lactone	Boronate<br>Carboxylic acid	Nitro<br>N-Fmoc-Amino<br>Carboxylic-acid
	Phenylalanine	Borane	Carboxylic-acid<br>Aryl-halide	Terminal-alkyne<br>N-Boc-Amino<br>Carboxylic-acid
	Tyrosine	Boronic acid	Carboxylic-acid Aryl-pseudohalide	
	Tryptophan	Boronic ester	Carboxylic-acid<br>Isothiocyanate	
	Aspartate	Carbamate	Carboxylic-acid Nitro	
	Glutamate	Cyanide	Carboxylic-acid<br>Terminal-alkyne	
	Histidine	Disulfide	Ester<br>Aldehyde	
	Lysine	Epoxide	Ester	

			Isocyanate	
	Arginine	Haloamide	Ester<br>Sulfonyl-halide	
	Selenocystei ne	Haloketone	N-Boc-Amino<br>Aldehyde	
	Pyrrolysine	Isothiocyanate	N-Boc-Amino<br>Carboxylic-acid	
		Ketoamide	N-Cbz-Amino<br>Carboxylic-acid	
		Maleimide	Sulfonyl-halide<br>Aldehyde	
		Oxaziridine		
		Phosphonates		
		Propiolonitrile		
		Sulfonimidoyl fluoride		
		Sulfonyl ester		
		Sulfonyl halide		
		Michael acceptor		
		Vinyl sulfonamide		
		Vinyl sulfone		

Table S2. Functional groups or substructures required for the corresponding DIY focused subsets.

ARCHIE (enumeration algorithm by Mcule, version 2022) details

The algorithm used in the study to build the DIY virtual library is ARCHIE (Mcule, version: 2022). It is a command line tool for enumerating combinatorial virtual libraries from a set of building blocks and reaction SMARTS rules. ARCHIE was used by default settings except that from all the available reaction rules, four major reaction types (amide formation, ester bond formation, catalytic carbon-carbon coupling and reactions of heteroaromatic halides and nucleophiles) were selected as main reactions. The algorithm is fed by 4 different files:

1. The first input file should contain the main reactions as reaction SMARTS patterns indicating the reacting atoms which are involved in bond formation or cleavage. These patterns define the allowed environment around the reaction centre to ensure high reactivity.
2. The second file contains the side reactions as SMARTS patterns. These reaction sides are checked within the building blocks and if there is a match for a competitive site then the building block is skipped and will not be able to react. Most important examples include:

- Acylations (amidations, ester formations, sulfonamide formations, etc.)
- Catalytic couplings (palladium-, copper catalyzed reactions, etc.)
- Heterocycle forming reactions (e.g. oxadiazoles, triazoles, quinolines, etc.)
- Alkylations (Williamson ethers, reductive aminations, thioether formations, etc.)

- Deprotections (Cbz, Fmoc, BOC, etc.)
- Aromatic nucleophilic substitutions

3. The third file defines the relationship between the main and side reactions. What side reaction should be taken into consideration next to each main reaction (e.g. when considering amide formation as a main reaction, a Suzuki- or Sonogashira coupling is not possible as a side reaction (due to the absence of the catalyst), while an ester formation or an amine alkylation with an alkyl halide could occur, as the required reagents for these are present in the reaction mixture).
4. The fourth file contains the input building blocks.

The speed of the enumeration strongly depends on the number and complexity of the main and side reactions. In the current scenario, the speed of enumeration was 134k products / min on a 24 core CPU (AMD EPYC™ 7401P) thus the enumeration of the complete 14 million DIY library required only 105 minutes.

Reaction validations

Amide formation

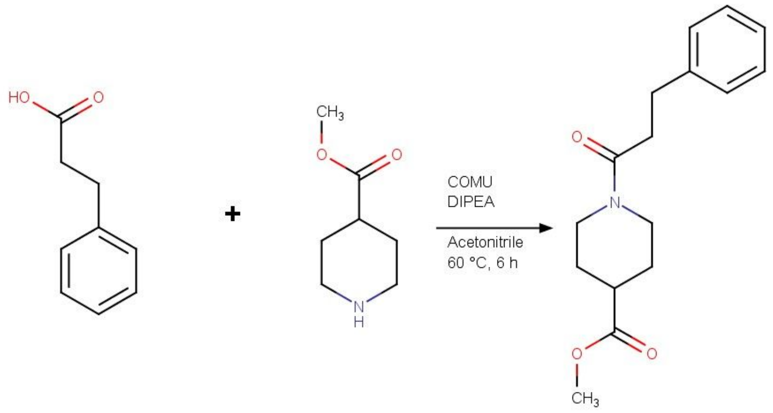

Figure S1. Reaction scheme of amide formation

Methyl 1-(3-phenylpropanoyl)piperidine-4-carboxylate was prepared by the reaction between 19.5 mg (0.130 mmol) methyl-piperidine-4-carboxylate (BLDPharm, BD3901) and 21.6 mg (0.151 mmol) 3-phenylpropanoic acid (A2B Chem LLC, AB74711). The reactants were dissolved in acetonitrile (750 μL) and 57.7 mg (1-cyano-2-ethoxy-2-oxoethylidenaminooxy)dimethylamino-morpholino-carbenium hexafluorophosphate (COMU - 0.135 mmol)[1], 28 μL N,N-diisopropylethylamine (DIPEA - 0.156 mmol) were added to the solution.[2] The mixture was stirred at 60 °C for 6 hours. The final product was obtained via preparative HPLC. Isolated product was found to be 100% pure, 3.2 mg was isolated, the spectra are shown in figures S2 and S3.

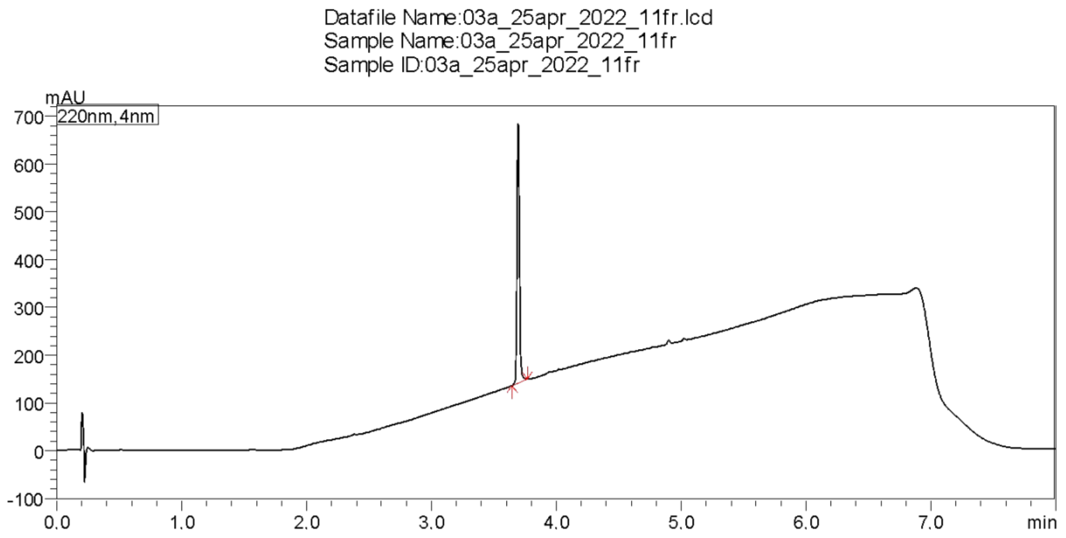

Figure S2. Spectrum of purified amide formation product, retention time of target compound: 3.7 min

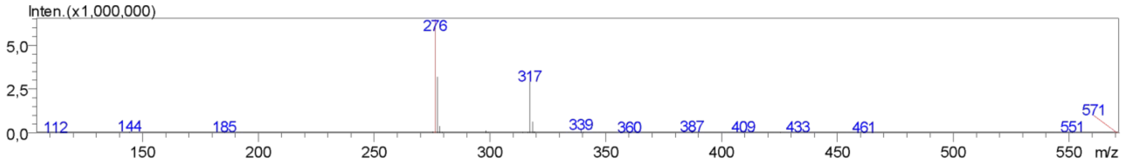

Figure S3. Mass spectrum of target compound, product ion (M+H+) at 276 m/z

Aromatic nucleophilic substitution reaction

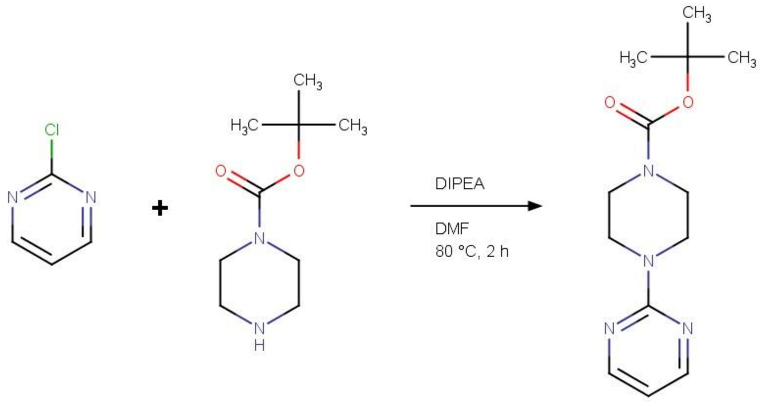

Figure S4. Reaction scheme of SNAr reaction

Tert-butyl 4-(pyrimidin-2-yl)piperazine-1-carboxylate was prepared by the reaction between 1.9 mg (0.0167 mmol) of 2-chloropyrimidine (BLDPharm, BD9389) and 1.8 mg (0.0197 mmol) of tert-butyl piperazine-1-carboxylate (Angene, AG0035MU). The reactants were dissolved in 400 µL dimethylformamide (DMF) and 9 µL of N,N-diisopropylethylamine (0.0517 mmol) was added.[3] The mixture was stirred at 80 °C for 2 hours. The reaction mixture was then purified directly via preparative HPLC to achieve 0.3 mg of isolated product with 94% purity.

Purity was determined by LCMS, where the McLafferty-rearrangement of *tert*-butoxycarbonyl (BOC) protection group was observed due to ESI-MS analysis, the reaction scheme is shown on Figure S5. As indicated by Wolf et al., this process only occurs during ionization, but the structure is still stable.[4]

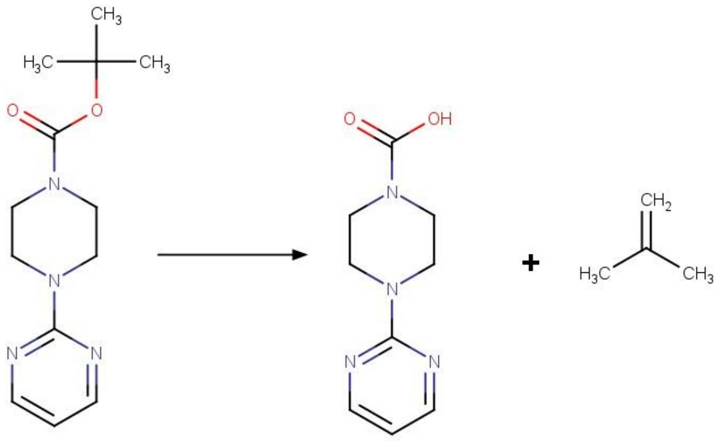

Figure S5. McLafferty-rearrangement occurring during ionization - target product mass 264 g/mol, rearrangement product mass 208 g/mol

Spectra of the S_NAr reaction product are shown in figures S6 and S7.

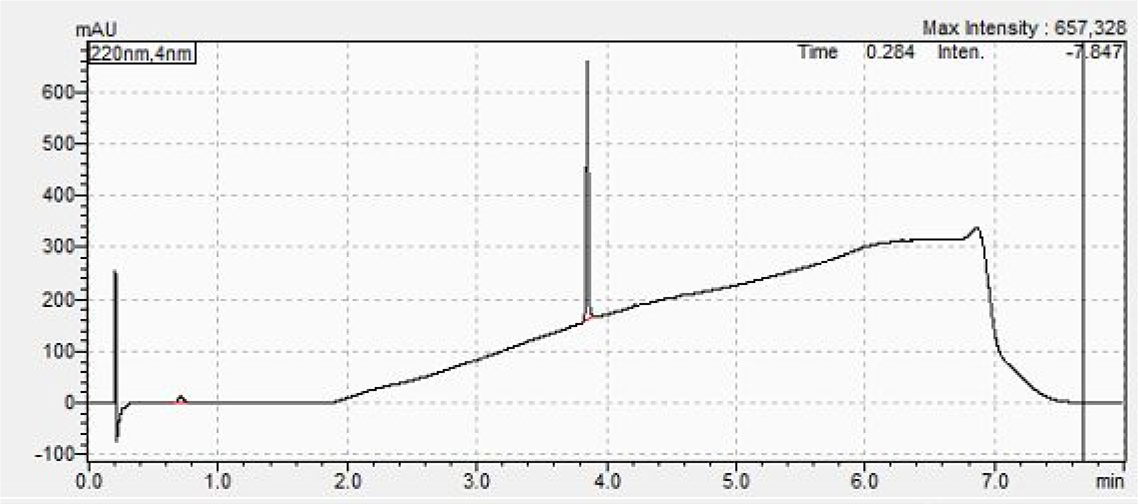

Figure S6. Spectrum of purified SNAr reaction product, retention time of target compound: 3.8 min

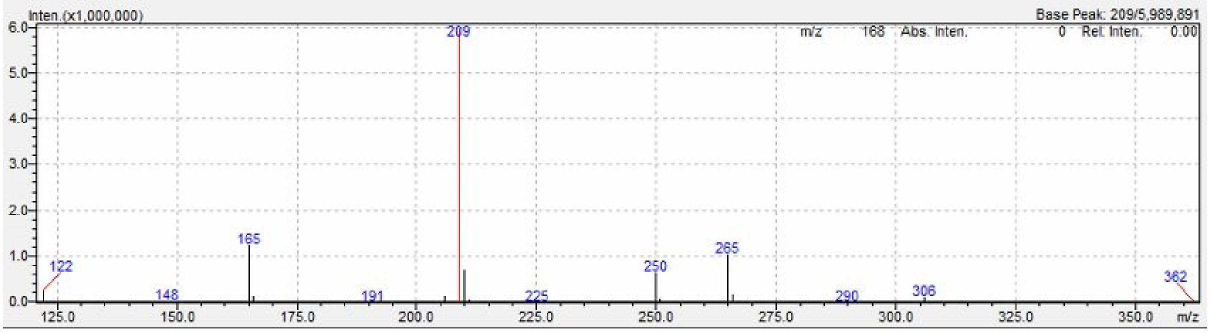

Figure S7. Mass spectrum of the purified product - product ion at 265 m/z (M+H$^+$), McLafferty-rearrangement product at 209 m/z ([M-56]+H$^+$)

*To confirm that not the reaction conditions cause the observed rearrangement, the same reaction was also performed in 600 μL dimethylsulfoxide (DMSO) in the presence of 12.0 mg of K_3PO_4, using 6.0 mg of 2-chloropyrimidine and 5.0 mg of tert-butyl piperazine-1-carboxylate[5], where the final product could be isolated similarly (observing the McLafferty-rearrangement as well).

2-step synthesis with ester formation and Suzuki cross coupling

Methyl 4-(3-{[(pyridin-2-ylcarbonyl)oxy]methyl}phenyl)pyridine-2-carboxylate was prepared in two separate reaction steps.

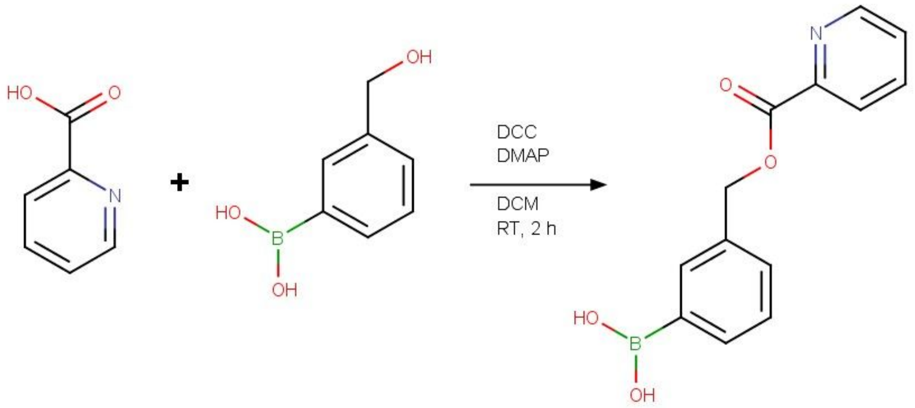

Figure S8. Ester formation reaction scheme

{3-[(pyridine-2-carbonyloxy)methyl]phenyl}boronic acid was prepared from [3-(hydroxymethyl)phenyl]boronic acid (BLDPharm, BD8123) and pyridine-2-carboxylic acid (BLDPharm, BD17192).

76.8 mg of pyridine-2-carboxylic acid (0.624 mmol) was dissolved in dichloromethane. For the solution 1.2 eq. of dicyclohexyl carbodiimide (DCC) was added as a coupling agent and 1.5 eq. of 4-dimethylaminopyridine (DMAP) as a base was added.[5,6] The mixture was stirred for 30 min. and 77.2 mg [3-(hydroxymethyl)phenyl]boronic acid (0.508 mmol), dissolved in dichloromethane was added to the reaction mixture. The mixture was stirred at room temperature (24 °C) for 2 hours in a closed vial. The final product mixture was evaporated and purified via preparative thin-layer chromatography.

53.0 mg of {3-[(pyridine-2-carbonyloxy)methyl]phenyl}boronic acid could be isolated and confirmed by LCMS.

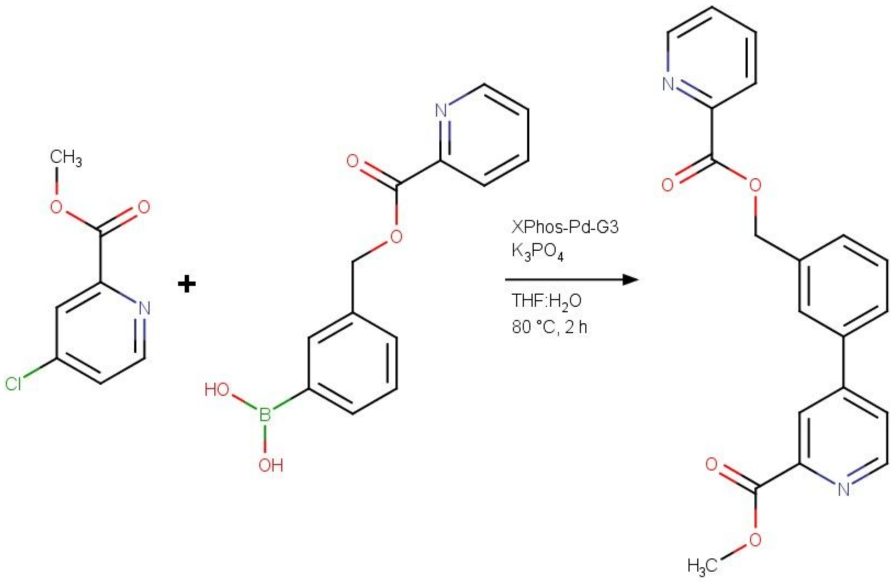

Figure S9. Reaction scheme of Suzuki coupling

The obtained 53.0 mg (0.206 mmol) of {3-[(pyridine-2-carbonyloxy)methyl]phenyl}boronic acid was dissolved in tetrahydrofuran-water (2:1) and 20.4 mg of methyl 4-chloropyridine-2-carboxylate (0.119 mmol) (BLDPharm, BD19625) was added to the solution. The mixture was degassed and purged with argon. XPhos-Pd-G3[7] (catalyst-ligand system) (2 %) and K_3PO_4 (base) (0.2 M) was added and the reaction mixture was sealed, stirred for 2 hours at 80 °C on a magnetic stirrer equipped with a heating block. The final mixture was evaporated and purified by preparative thin-layer chromatography, to achieve an isolated amount of 2.1 mg methyl 4-(3-{[(pyridin-2-ylcarbonyl)oxy]methyl}phenyl)pyridine-2-carboxylate, the spectra are shown in Figures S10 and S11.

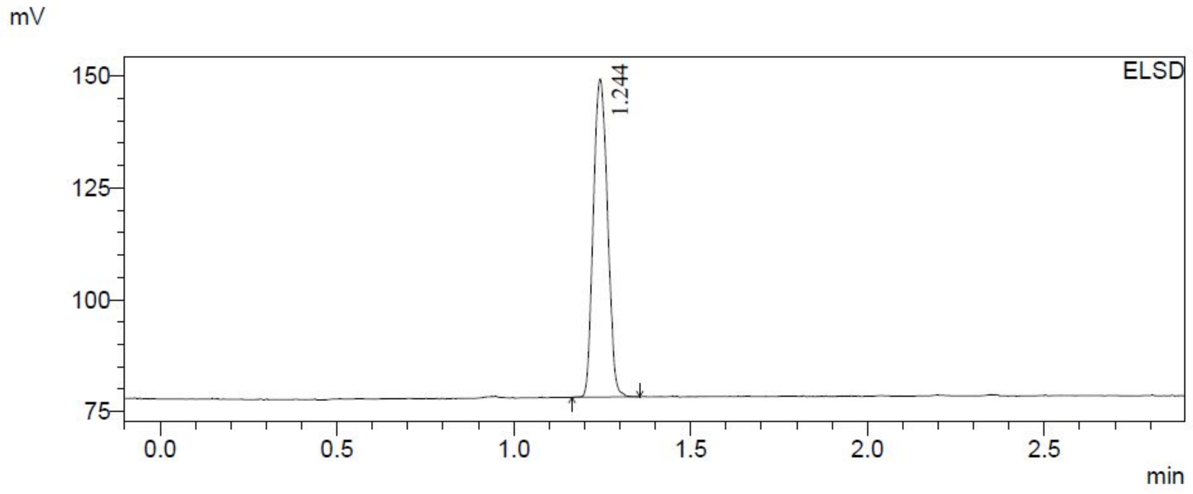

Figure S10. Spectrum of purified 2-step reaction product, retention time of target compound: 1.2 min

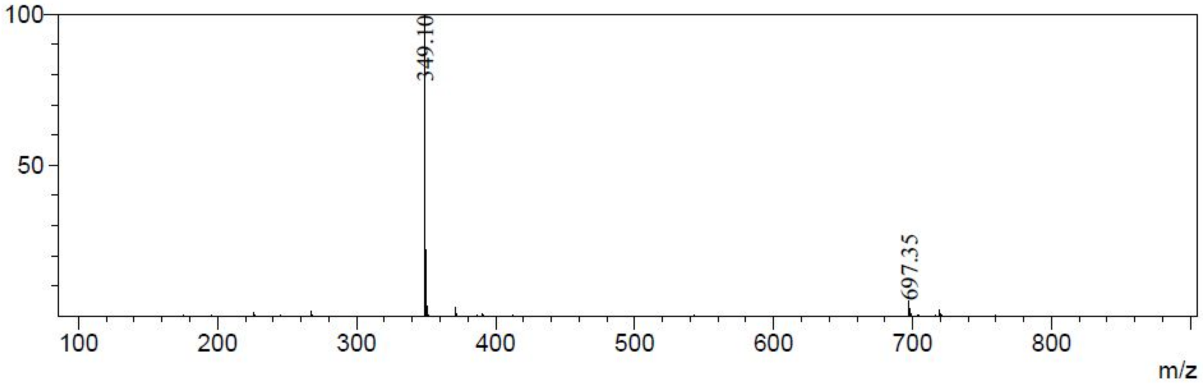

Figure S11. Mass spectrum of target compound, product ion at 349 m/z (M+H+), dimer ion of product at 697 (M+M+H+)

2-step synthesis with SNAr and ester formation

2-(1H-imidazol-1-yl)ethyl 1-(5-nitropyridin-2-yl)piperidine-4-carboxylate was prepared in two separate reaction steps.

10 mg (0.063 mmol) of 2-chloro-5-nitropyridine (BLDPharm: BD2544) and 10 mg (0.077 mmol) of piperidine-4-carboxylic acid (Angene: AG00351Y) were dissolved in 500 uL of dimethyl formamide, and 18 uL DIPEA (0.103 mmol) were added. The reaction mixture was stirred at 90 °C for 1.5 h. With preparative HPLC, 9 mg final product (1-(5-nitropyridin-2-yl)piperidine-4-carboxylic acid) was isolated, with 94.3% purity by UV.

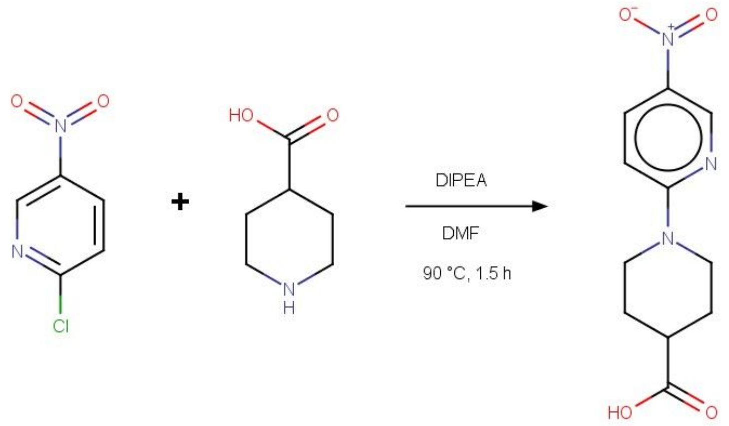

Figure S12. Reaction scheme of S_NAr reaction

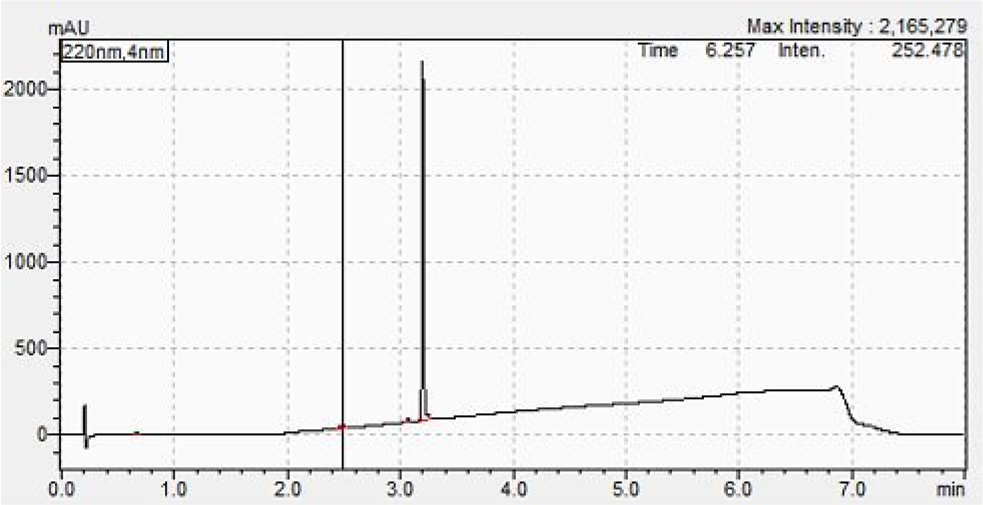

Figure S13: Spectrum of purified intermediate, retention time of target at 3.2 min

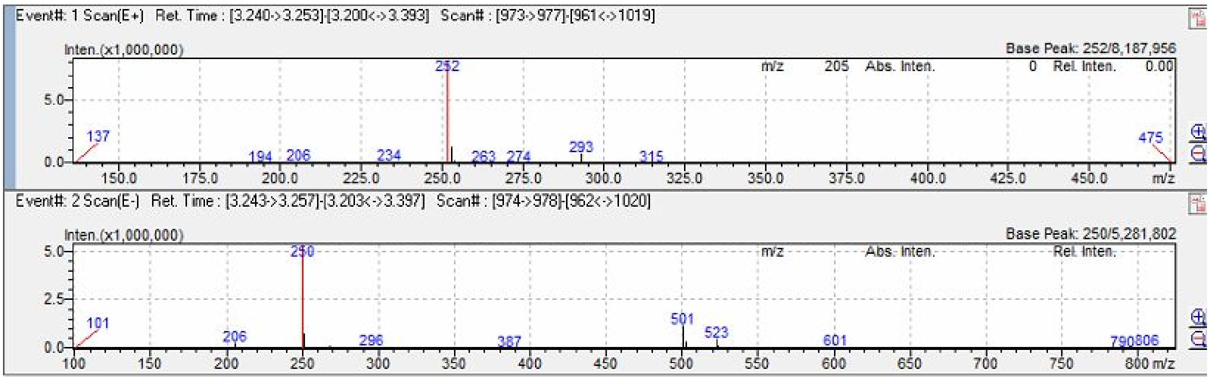

Figure S14: Mass spectrum of target compound, product ion at 252 m/z (M+H+) in positive mode, 250 m/z (M-) in negative mode

9 mg (0.036 mmol) of 1-(5-nitropyridin-2-yl)piperidine-4-carboxylic acid, 14.4 mg (0,128 mmol) of 2-(1H-imidazol-1-yl)ethanol (A2B Chem LLC: AB50478), 13,6 mg (0.043 mmol) fluoro-N,N,N′,N′-bis(tetramethylene)formamidinium hexafluorophosphate (BTFFH) and 9 uL (0.052 mmol) DIPEA were dissolved in 400 uL of acetonitrile.[8] The reaction mixture was stirred at 60 °C for 1.5 h. 12.1 mg of 2-(1H-imidazol-1-yl)ethyl 1-(5-nitropyridin-2-yl)piperidine-4-carboxylate was isolated by preparative HPLC, with 92.9% purity by UV.

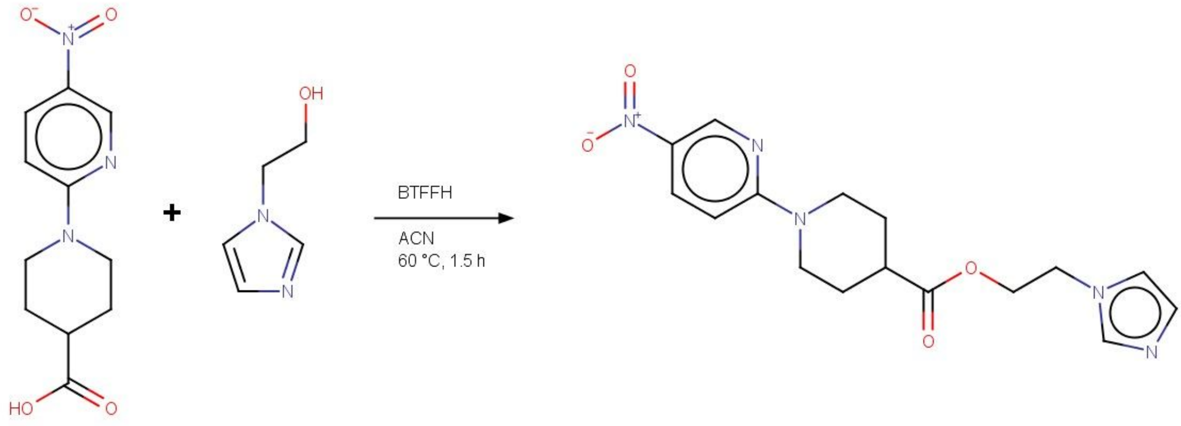

Figure S15. Reaction scheme of ester formation

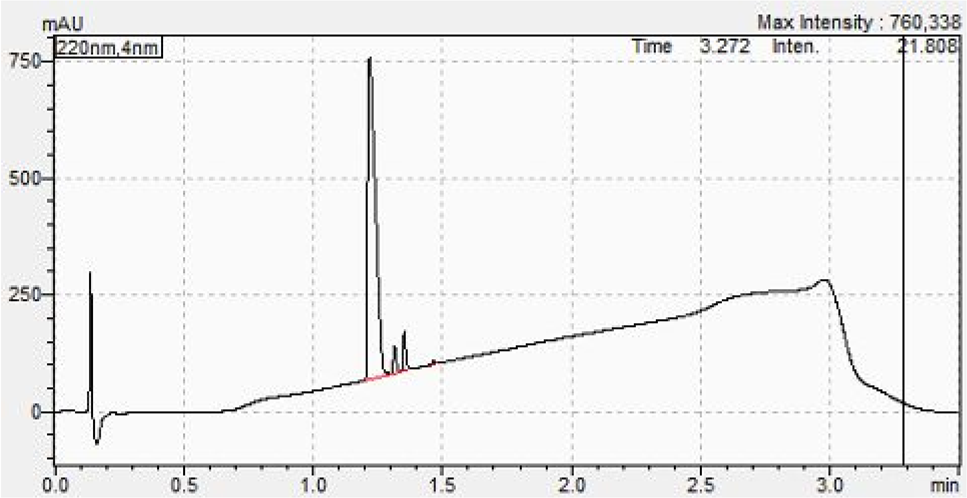

Figure S16: Spectrum of purified product, retention time of target at 1.2 min

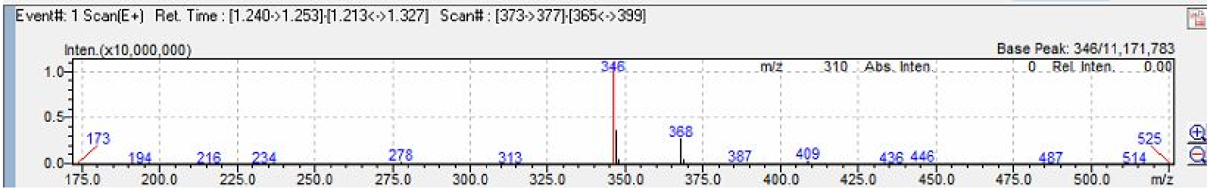

Figure S17: Mass spectrum of target compound, product ion at 346 m/z (M+H$^+$) in positive mode

2-step synthesis with Suzuki coupling and amide formation

2-[6-(4-tert-butylphenyl)pyridin-3-yl]-1-(3,4-dihydro-2H-quinolin-1-yl)ethanone was prepared in two separate reaction steps.

20.7 mg (0.116 mmol) of 4-tert-Butylphenylboronic acid (BLDPharm: BD0263) and 19.2 mg (0.112 mmol) of (6-chloropyridin-3-yl)acetic acid (A2B Chem LLC: AI49749) with 37,3 mg Cs2CO3, 0.7 mg N,N′-bis(2,4,6-trimethylphenyl)ethane-1,2-diamine palladium(II) dibromide (Pd(BEDA)Cl_2) in 525 uL IPA:water 20:1, for 1.5 h at 90 °C.[9] 3.1 mg [6-(4-tert-butylphenyl)pyridin-3-yl]acetic acid isolated by preparative HPLC with 100% purity by UV.

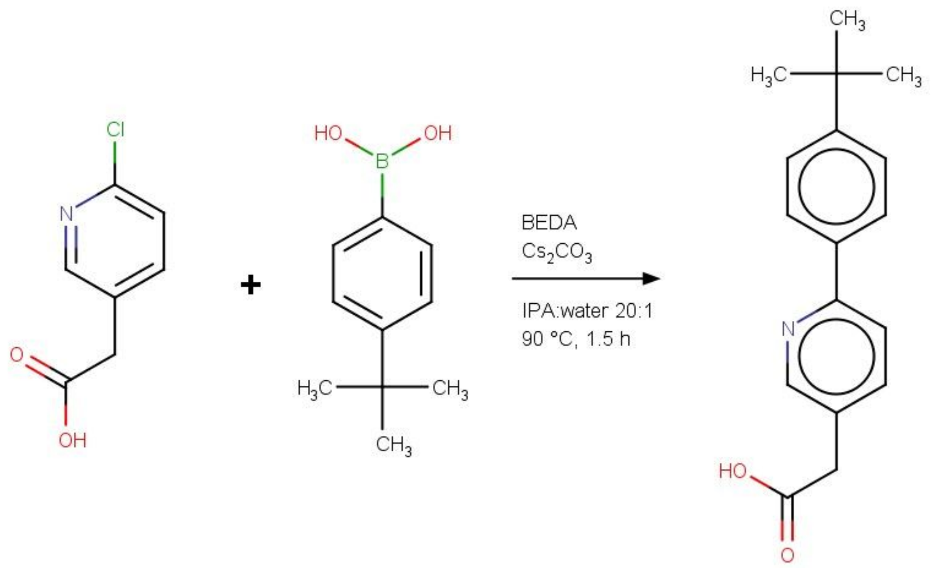

Figure S18. Reaction scheme of Suzuki coupling

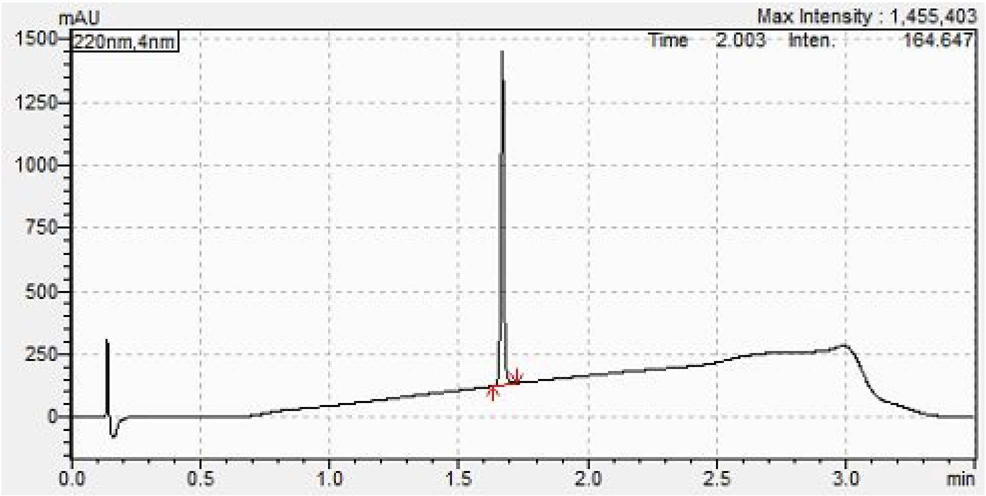

Figure S19: Spectrum of purified intermediate, retention time of target at 1.6 min

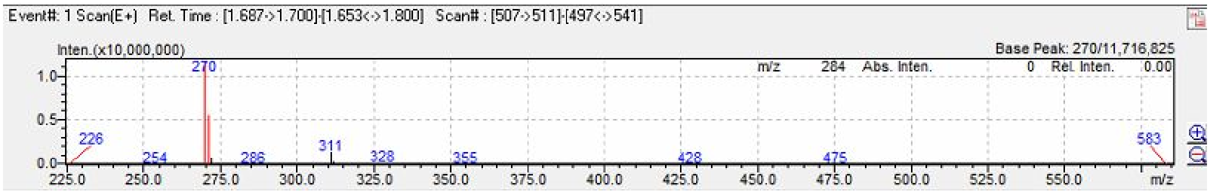

Figure S20: Mass spectrum of purified intermediate, product ion at 270 m/z ($M+H^+$) in positive mode

3.1 mg (0.016 mmol) [6-(4-tert-butylphenyl)pyridin-3-yl]acetic acid, 4.0 mg (0.030 mmol) of 1,2,3,4-tetrahydroquinoline (Angene: AG003DAA), 5.3 mg (0.014 mmol) HATU and 3 uL DIPEA were added to 300 uL acetonitrile. The mixture was stirred at 60 °C for 1.5 h. 2.1 mg of 2-[6-(4-tert-butylphenyl)pyridin-3-yl]-1-(3,4-dihydro-2H-quinolin-1-yl)ethanone was isolated with preparative HPLC, with 96.3% purity by UV.

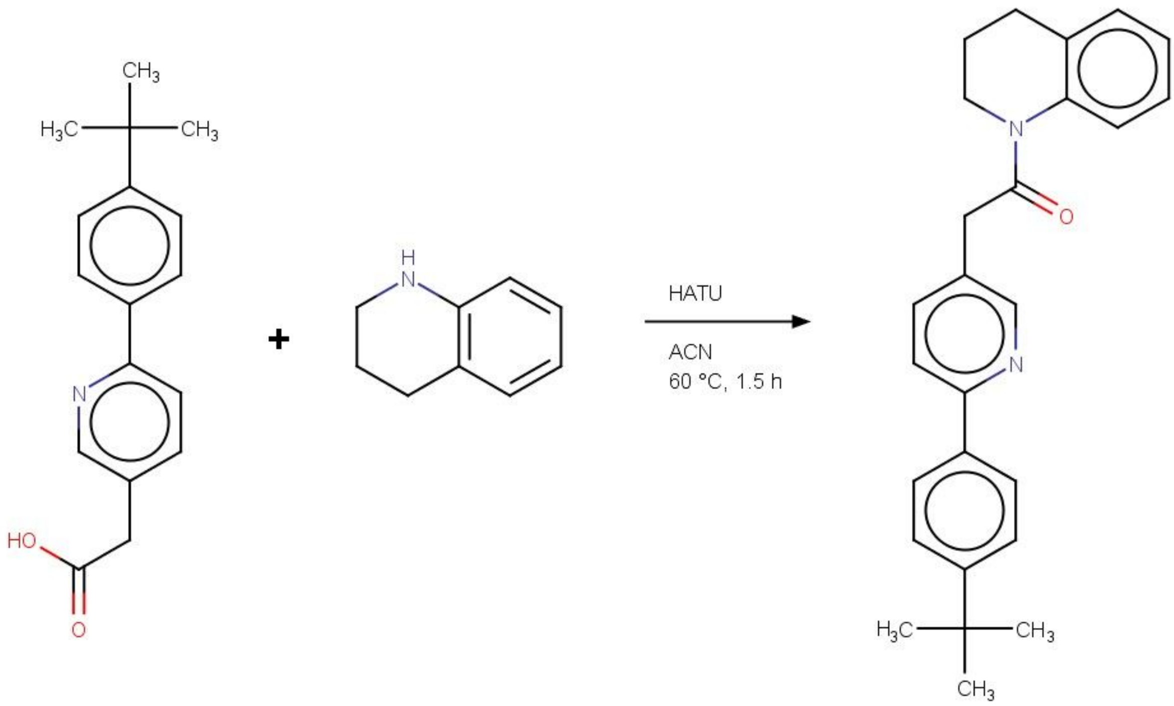

Figure S21. Reaction scheme of amide formation

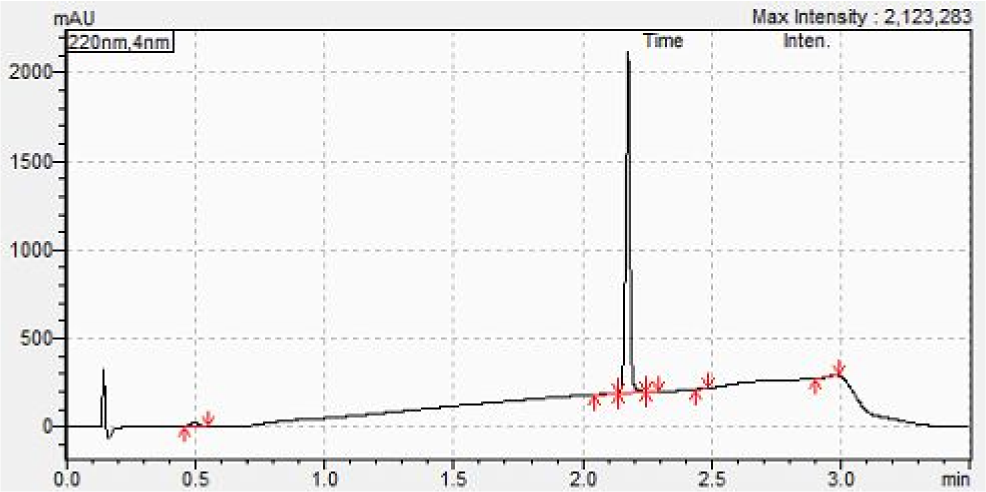

Figure S22: Spectrum of purified product, retention time of target at 2.2 min

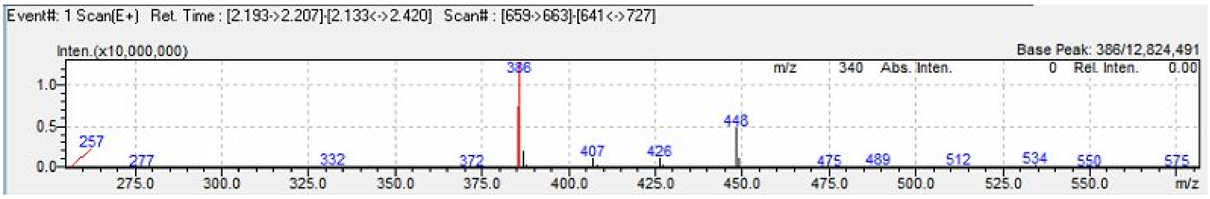

Figure S23: Mass spectrum of target compound, product ion at 386 in positive mode

2-step synthesis with Sonogashira coupling and SNAr

2-[4-[4-[2-(4-ethoxyphenyl)ethynyl]pyridin-2-yl]piperazin-1-yl]pyrimidine was prepared in two separate reaction steps.

13.2 mg (0.059) 2-fluoro-4-iodopyridine (BLDPharm: BD17172) and 15.0 mg (0.103) 1-ethoxy-4-ethynylbenzene (Angene: AG003LBI), mg (0.003) of bis(triphenylphosphine)palladium(II) dichloride ($Pd(TPP)_2Cl_2$), 0.8 mg of $CuSO_4$ and 2.1 mg of sodium ascorbate (0.011) were added to 400 uL of triethylamine : acetonitrile 1:3 mixture and stirred for 1 h at 90 °C.[10] 10 mg of 4-[(4-ethoxyphenyl)ethynyl]-2-fluoropyridine was isolated by preparative HPLC with 98.2% purity.

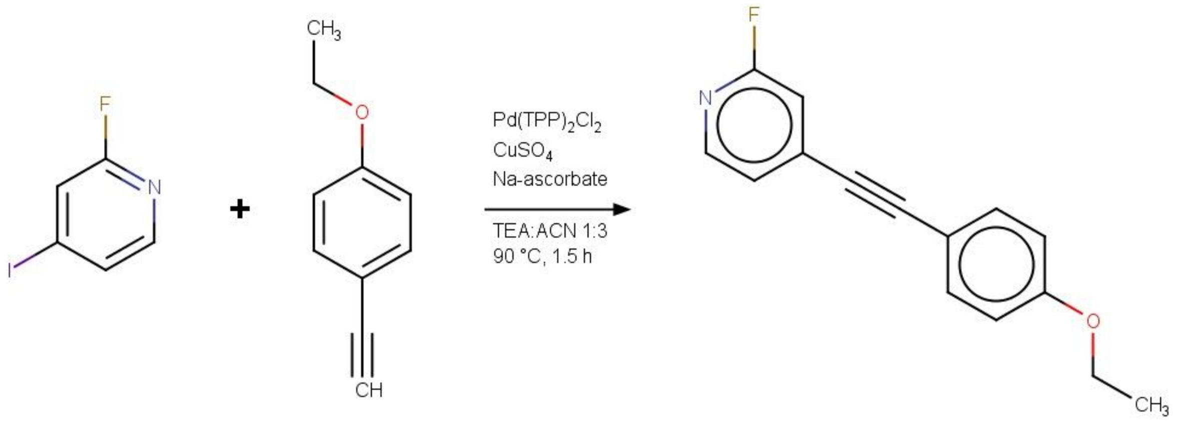

Figure S24. Reaction scheme of Sonogashira coupling

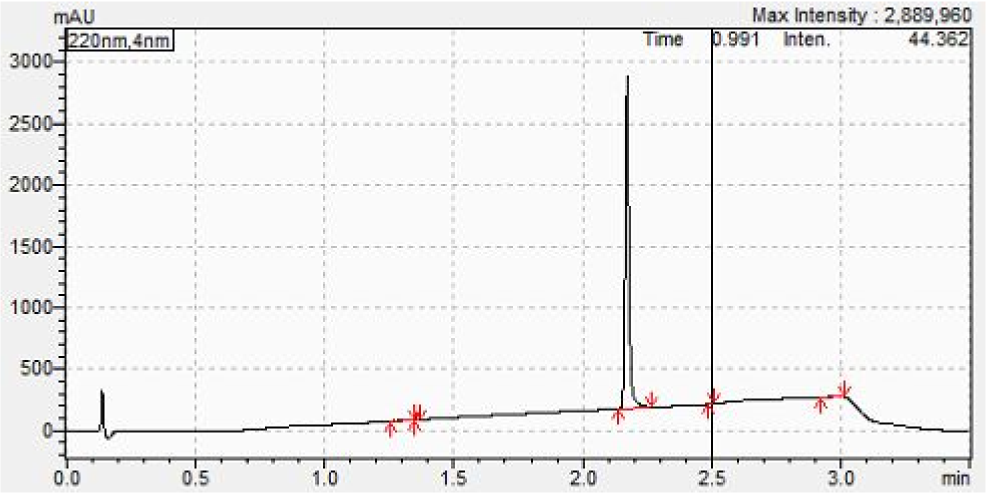

Figure S25: Spectrum of purified intermediate, retention time of target at 2.2 min

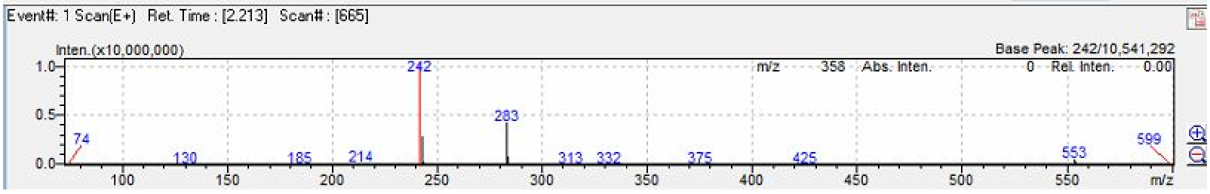

Figure S26: Mass spectrum of intermediate, product ion at 242 m/z (M+H+) in positive mode

5.1 mg (0.021 mmol) 4-[(4-ethoxyphenyl)ethynyl]-2-fluoropyridine, 5.1 mg (0.031 mmol) 2-piperazin-1-ylpyrimidine (Angene: AG002KF5) in 400 uL of DMF in the presence of 6 uL (0.034 mmol) DIPEA. The reaction was stirred at 90 °C for 16 h. 0.8 mg of 2-[4-[4-[2-(4-ethoxyphenyl)ethynyl]pyridin-2-yl]piperazin-1-yl]pyrimidine was isolated by preparative HPLC with 91.6% purity.

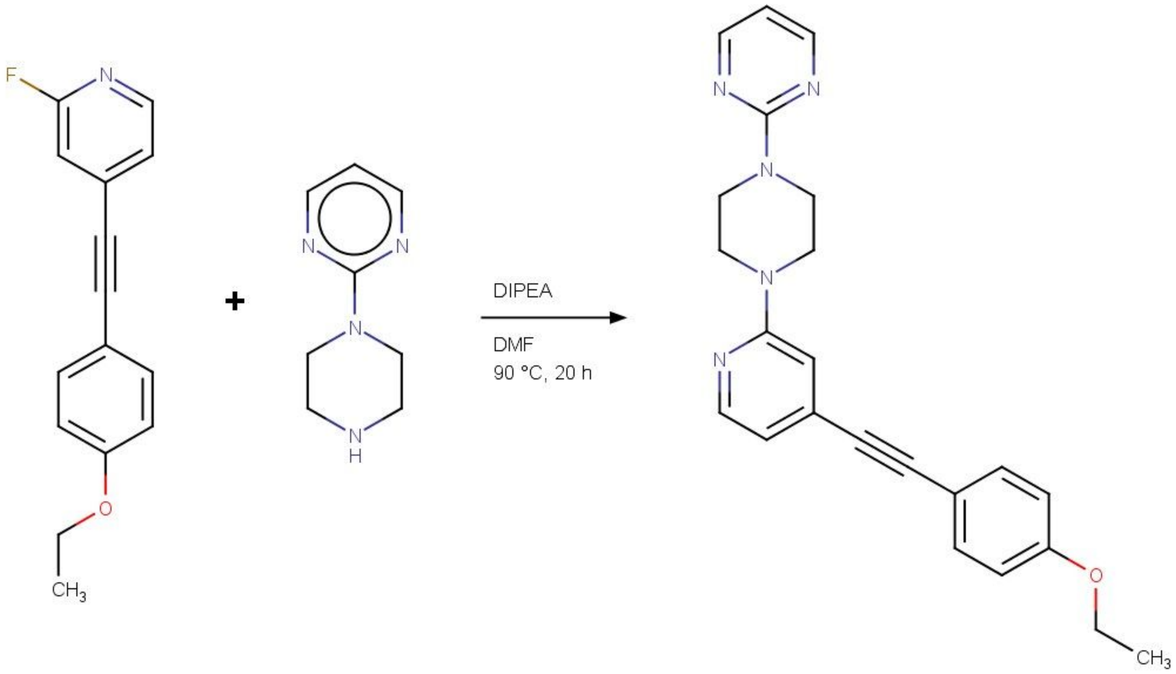

Figure S27. Reaction scheme of S_NAr reaction

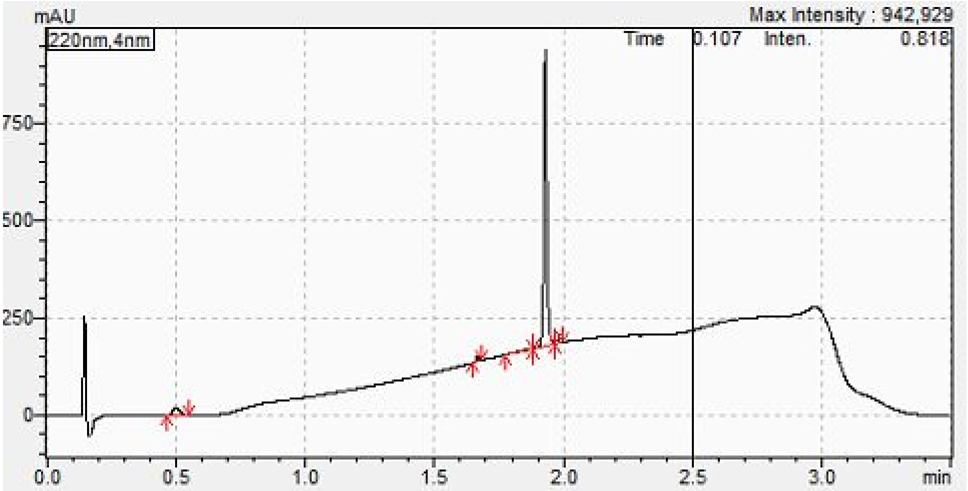

Figure S28: Spectrum of purified product, retention time of target at 1.9 min

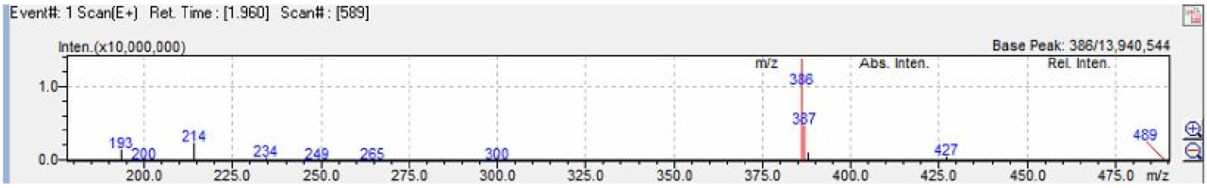

Figure S29: Mass spectrum of purified product, product ion at 386 m/z (M+H$^+$) in positive mode

No unexpected or unusually high safety hazards were encountered.

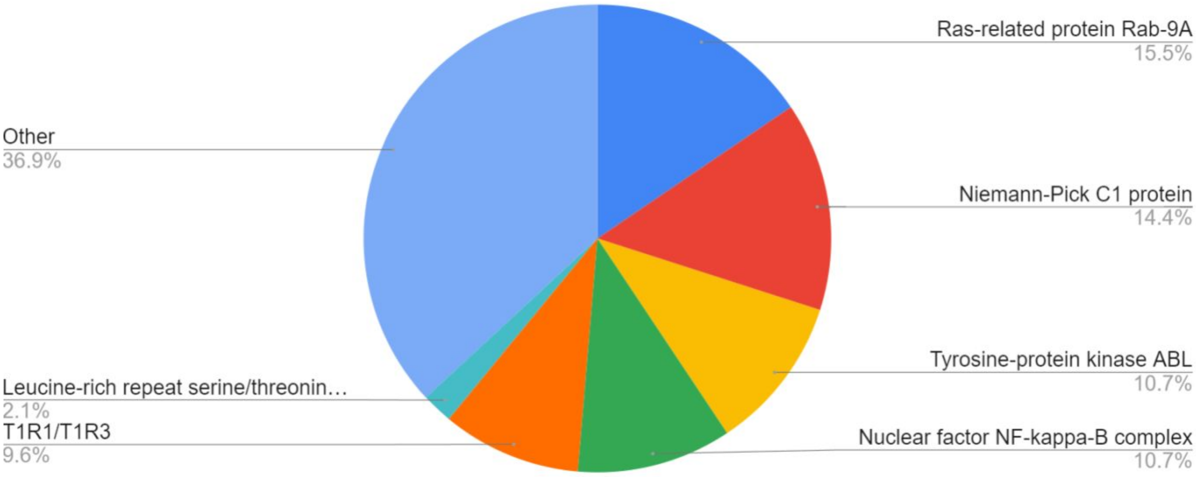

Figure S30. The ratio of reported activities of the 88 reported bioactive compounds of the library.

(1) El-Faham, A.; Albericio, F. COMU: A Third Generation of Uronium-Type Coupling Reagents. *Journal of Peptide Science*. **2010**, pp 6–9. https://doi.org/10.1002/psc.1204.

(2) Kumar, A.; Jad, Y. E.; de la Torre, B. G.; El-Faham, A.; Albericio, F. Re-Evaluating the Stability of COMU in Different Solvents. *J. Pept. Sci.* **2017**, *23* (10), 763–768.

(3) Abdildinova, A.; Kurth, M. J.; Gong, Y.-D. Heterocycles as a Peptidomimetic Scaffold: Solid-Phase Synthesis Strategies. *Pharmaceuticals* **2021**, *14* (5). https://doi.org/10.3390/ph14050449.

(4) Wolf, C.; Villalobos, C. N.; Cummings, P. G.; Kennedy-Gabb, S.; Olsen, M. A.; Trescher, G. Elucidation of the Presence and Location of T-Boc Protecting Groups in Amines and Dipeptides Using on-Column H/D Exchange HPLC/ESI/MS. *J. Am. Soc. Mass Spectrom.* **2005**, *16* (4), 553–564.

(5) Kikushima, K.; Koyama, H.; Kodama, K.; Dohi, T. Nucleophilic Aromatic Substitution of Polyfluoroarene to Access Highly Functionalized 10-Phenylphenothiazine Derivatives. *Molecules* **2021**, *26* (5). https://doi.org/10.3390/molecules26051365.

(6) Neises, B.; Steglich, W. Simple Method for the Esterification of Carboxylic Acids. *Angewandte Chemie International Edition in English*. **1978**, pp 522–524. https://doi.org/10.1002/anie.197805221.

(7) Bruno, N. C.; Tudge, M. T.; Buchwald, S. L. Design and Preparation of New Palladium Precatalysts for C–C and C–N Cross-Coupling Reactions. Chem. Sci. 2013, 4, 916–920.

(8) Due-Hansen, M. E.; Pandey, S. K.; Christiansen, E.; Andersen, R.; Hansen, S. V. F.; Ulven, T. A protocol for amide bond formation with electron deficient amines and sterically hindered substrates. Org. Biomol. Chem. 2016, 14, 430-433.

(9) Liu, G.; Han, F.; Liu, C.; Wu, H.; Zeng; Y.;Zhu, R.; Yu, X.; Rao, S.; Huang, G.; Wang, J. A Highly Active Catalyst System for Suzuki–Miyaura Coupling of Aryl Chlorides. Organometallics 2019, 38, 1459-1467.

(10) Wang, D.; Gao, S. Sonogashira Coupling in Natural Product Synthesis. Org. Chem. Front. 2014, 1, 556-566.
